# Unraveling the dynamics of wheat leaf blight complex: isolation, characterization, and insights into pathogen population under Indian conditions

**DOI:** 10.3389/fmicb.2024.1287721

**Published:** 2024-02-21

**Authors:** Sanghmitra Aditya, Rashmi Aggarwal, Bishnu Maya Bashyal, Malkhan Singh Gurjar, Mahender Singh Saharan, Shweta Aggarwal

**Affiliations:** Fungal Molecular Biology Laboratory, Division of Plant Pathology, ICAR-Indian Agricultural Research Institute, New Delhi, India

**Keywords:** leaf blight/spot blotch complex, wheat, soil population dynamics, absolute quantification, *Bipolaris spicifera*, *Exserohilum rostratum*

## Abstract

Wheat, a staple food crop for 35% of the global population, faces a threat from Helminthosporium leaf blight (HLB), a complex of spot blotch (*Bipolaris sorokiniana*) and tan spot (*Pyrenophora-tritici-repentis*) diseases under warm and humid conditions. However, in Indian conditions, the knowledge of existing pathogen populations associated with the HLB complex is limited and largely dominated by only *B. sorokiniana* (spot blotch). To address this, diseased samples were collected from all six wheat growing zones during 2020–2022. The pathogenic species were identified through in-depth morphological characterization, supplemented with ITS-rDNA and GAPDH sequence analysis, a diagnostic SCAR marker, and pathogenicity studies on two wheat varieties: Sonalika and HD2733. The 32 isolates collected from 10 different states consist of *B. spicifera* (12.5% of all isolates)*, Exserohilum rostratum* (9.3%)*, Bipolaris oryzae* (3.1%), and *B. sorokiniana* (75%)*. B. sorokiniana* exhibited the highest disease severity on both varieties. Other lesser-known pathogenic species also produced comparable disease severity as *B. sorokiniana* isolates and, therefore are economically important. Unraveling pathogen composition and biology aids in disease control and resistance breeding. Our study highlights economically impactful and lesser-known pathogenic species causing wheat leaf blight/spot blotch in India, guiding both current management and future resistance breeding strategies in plant pathology.

## 1 Introduction

Wheat supports 35 percent of the world’s population and contributes to 20 percent of the daily allowance of calories and proteins; thus, it holds the title of “the king of cereals.” According to Food and Agriculture Organization (FAO) estimates for the year 2022, 770 million metric tons of wheat are produced on 221 million hectares of area worldwide (FAOSTAT, 2022).^1^ Despite these massive production statistics, wheat production must expand to satisfy the anticipated global food demand of approximately 9 billion people by 2050 (CIMMYT, 2013).^2^ India is the world’s second-largest producer of wheat, accounting for 13.53 percent of global output. It provides 50 percent of daily calories and the highest *per capita* per day gain of micronutrients such as iron (7.45 mg) and zinc (5.6 mg), as such contributing the most to nutritional security in India (Grewal and Goel, 2015; IIWBR, 2023).^3^ India’s ever-increasing population, placing immense pressure on the land, and the progressive shrinking of arable land (0.03 million hectares per year) create an urgent need to maximize wheat production. One of the major strategies for increasing production involves minimizing the yield losses caused by the various biotic and abiotic agents, with diseases playing a major role by causing an annual loss of 20 percent worldwide (Gurung et al., 2012; Khan et al., 2020).

One of the most destructive diseases of wheat leading to significant yield losses throughout the world is the Helminthosporium leaf blight (HLB) complex. The disease impacts crops in warm and humid areas of the world, especially South Asia with its intensive rice-wheat cropping system (He et al., 2022). It has become a major constraint in wheat production, particularly in the Indo-Gangetic Plains of Bangladesh (Siddique et al., 2006), India (Aggarwal et al., 2009; Devi et al., 2018; Aggarwal et al., 2021), Nepal (Pandey et al., 2018), and Pakistan (Iftikhar et al., 2009), as well as other parts of the world where wheat is cultivated (Acharya et al., 2011). The Eastern Gangetic Plain (EGP) in South Asia, with warm and humid weather, is considered a hotspot for the HLB complex (Sundeep et al., 2013). As a result of climate change, it is also becoming more prevalent in the North Western Plain Zone (NWPZ), which accounts for increased yield losses in susceptible varieties (Chowdhury et al., 2013; Sundeep et al., 2013; Kumar et al., 2020). A total of 25 million hectares around the world are affected by the disease (Sharma et al., 2007), about 40 percent of which is in the Indian subcontinent (Joshi et al., 2007). It is also prevalent in wheat-growing regions of Latin America (Poloni et al., 2009; Mann et al., 2014; Wu et al., 2021), Australia (Phan et al., 2019), West Asia (Arabi et al., 2019; Gholamaliyan et al., 2021), North Africa (Qostal et al., 2019; Karamian et al., 2022), sub-Saharan Africa (Tembo et al., 2017), Central Asia (Bozoğlu et al., 2022), and the great plains of the United States and Canada (Friesen et al., 2018; Manan et al., 2023).

The disease can cause more than 30 percent yield loss on susceptible cultivars, and under favorable climatic conditions, it can be devastating with up to 100 percent severity, resulting in complete yield loss in South Asian countries (Mehta, 1998; Sharma and Duveiller, 2004, 2007; Duveiller et al., 2005). The grain yield loss is reported up to 43 percent in Nepal (Sharma and Duveiller, 2006) and 22 percent in Bangladesh (Siddique et al., 2006). The disease also causes substantial damage in dryland environments, such as Syria (10–40%) (Arabi et al., 2019; Gholamaliyan et al., 2021) and Iran (3–12.5%) (Gholamaliyan et al., 2021; Karamian et al., 2022). In India, the disease reduces grain yields by 40–44 percent (Devi et al., 2018). The management option relies on seed treatment and applying two foliar sprays, the first upon the initiation of infection and the second 10–20 days after the first one. The triazole group of fungicides such as propiconazole, hexaconazole, tebuconazole, and triadimenol are effective in managing the disease. Also, carbendazim and azoxystrobin applications significantly reduce disease development (Sharma and Duveiller, 2006; Singh et al., 2014; Navathe et al., 2019).

HLB is a complex of spot blotch [induced by *Cochliobolus sativus* (Ito & Kuribayashi) Drechsler ex Dastur (anamorph: *Bipolaris sorokiniana* (Sacc.) Shoem.)] and tan spot [caused by *Pyrenophora tritici*-*repentis* (Died.) Drechsler (anamorph: *Drechslera tritici-repentis* (Died.) Shoem.)] (Duveiller et al., 2005). Under Indian conditions, *B. sorokiniana* is the predominant leaf blight/spot blotch pathogen associated with the HLB complex (Mehta, 1998; Gupta et al., 2018). It is a hemi-biotrophic pathogen that infects all parts of the plant and causes common root rot, spot blotch, and black point diseases. Spot blotch is primarily a seed-borne disease, but infection can also be initiated by inoculum surviving on crop residues, collateral hosts, resting mycelium, or conidia surviving in soil (Chand et al., 2002; Pandey et al., 2005). Although other pathogens have been found to be associated with the disease in the past, they are either non-pathogenic or do not cause substantial losses (Mehta, 1998), hence, they have never been well explored (Aggarwal et al., 2009; Bandyopadhyay et al., 2016; Aggarwal et al., 2022; Kashyap et al., 2023). The present study was conducted with the purpose of determining and comparing the extent of variability among the pathogens associated with the HLB/spot blotch complex, highlighting their current status in different wheat-growing zones of India. To achieve this, the study includes cultural, morphological, molecular, and pathogenicity characterization of all isolates. The survival and reproductive capacity of the associated pathogens in the rhizosphere of wheat varieties were also evaluated.

## 2 Materials and methods

### 2.1 Collection, isolation, and maintenance of fungal cultures

Spot blotch-infected leaf samples and black point infected seeds were collected from 10 different states in six wheat growing zones of India (the Central Zone, the North-Eastern Plain Zone, the Peninsular Zone, the South Hill Zone, the North Hill Zone, and the North-Western Plain Zone) during the *Rabi* season of 2020–22 (Supplementary Figure S1). Infected leaf samples were collected at the tillering to harvesting stage, and infected seeds were collected by selecting from seed stocks based on black point symptoms. Samples were immediately processed after reaching the laboratory. The leaf samples with distinct lesions were cut into bits of 2–5 mm squares and surface sterilized by dipping in 1 percent (v/v) sodium hypochlorite solution (NaOCl) (HiMedia, India) for 1 min, followed by three rinses in sterile distilled water. Similarly, the infected seeds were also cut, and the black tip at the embryonic region was taken and sterilized in a 2 percent sodium hypochlorite solution. The sterilized parts were then dried using sterilized filter paper and plated onto potato dextrose agar medium (HiMedia, India), four bits per plate with three replications for each sample. To avoid bacterial contamination, streptomycin sulfate was supplemented in the medium at a rate of 100 ppm. Petri plates were sealed with parafilm and incubated at 28 ± 2°C for 5 days in an alternate 12 h light/dark photoperiod. The mycelial growth was sub-cultured on fresh PDA plates using the hyphal tip technique and pure mono-conidial cultures were established through single spore isolation (Kumar et al., 2007; Aggarwal et al., 2009). After the establishment of pure cultures, they were subjected to morphological and molecular identification.

### 2.2 Morphological characterization

Morphological characterization of the colonies and spores of purified fungal isolates was performed. Colony morphology was assessed by placing a disc (1 cm in diameter) of purified fungal culture at the center of each plate (90 mm, Tarsons, India), sealing it with parafilm, and incubating it at 28 ± 2°C (Chaurasia et al., 2000). Observations on mycelium growth were taken at 3-day intervals until the 15^th^ day. Later on, the growth rate was calculated as a 15-day average of mean daily growth (mm/day). Colony pigmentation and mycelial color were observed on 7-day-old cultures and photographed (Nikon Coolpix P610, Japan). Furthermore, the cultures were grouped into distinct morphological classes according to Aggarwal et al. (2009). Spore morphology was assessed on 10-day-old cultures. Microscopic examination of conidia was carried out by washing the cultures in sterile water and observing the suspension under a phase contrast microscope (Nikon Eclipse Ci) with a drop of lactophenol (10x and 40x magnifications). Around 30–40 conidia per isolate were assessed for morphological variations in color, shape, size, and septation (Aggarwal et al., 2009). NIS-Elements Imaging software was used to measure the length and width of spores. For the determination of sporulation density, a disc (1 cm diameter) from a pure culture plate was carefully placed at the center of a fresh PDA plate and incubated at 28°C for 15 days. After 15 days, the plates were flooded with 5 mL of sterile distilled water and conidia were dislodged using a small brush. The suspension was homogenized by vigorous shaking in a vortex shaker (Vortex-Genie® 2 Shaker, Mumbai, India) for 10 min (Lee et al., 2021). A drop of filtered spore suspension was placed on the hemocytometer (Bright-Line™, Hemacytometer, Darmstadt, Germany) and spores were counted under the microscope (Nikon Eclipse Ci, Japan). All observations were taken with three replicates.

### 2.3 Molecular characterization

#### 2.3.1 Fungal DNA isolation

All the fungal cultures were grown in potato dextrose broth (HiMedia, India) at 28°C for 10 days in a shaker incubator (Kuhner, Switzerland). After 10 days, mycelial mats were harvested using Whatman filter paper in an aseptic environment and stored at −20°C until further processing. The lyophilized mycelial mat (100 mg) was ground into a fine powder with liquid nitrogen using a sterile pestle and mortar, and the CTAB (Cetyl trimethyl ammonium bromide) method was followed to extract the genomic DNA (Cullings, 1992). The extracted DNA was first assessed for quality and purity on 0.8 percent agarose gel (Green and Sambrook, 2019), followed by quantification on a Nanodrop spectrophotometer (Thermo Fisher Scientific Inc. 2000, United States). After the quality check, genomic DNA was stored at −20°C (Celfrost upright freezer, India) until further use.

#### 2.3.2 PCR amplification

The extracted DNA was subjected to molecular identification using the SCRABS_600_ marker, Internal Transcribed Spacer region (ITS), and protein-coding gene glyceraldehyde-3-phosphate dehydrogenase (GAPDH) sequencing. The PCR reaction was performed with DreamTaq Green PCR Master Mix (2X) (Thermo Fisher Scientific Inc., United States) by following the manufacturer’s instructions (40 ng of gDNA, 12.5 μL of DreamTaq Green PCR Master Mix (2X), 4 mM MgCl_2_, 2 μM of each primer in a final volume of 25 μL). Amplifications were performed on a T100^™^ Thermal cycler machine (Bio-RAD, United States).

#### 2.3.3 SCAR marker amplification

The SCAR marker, developed by Aggarwal et al. (2011), is unique to *B. sorokiniana* and specifically produces a distinct 600 bp band with *B. sorokiniana* DNA. The primers were synthesized (RABSF1: GGTCCGAGACAACCAACAA and RABSR2: AAAGAAAGCGGTCGACGTAA) from Agrigenome Labs Pvt. Ltd. (India) as described by Aggarwal et al. (2011). The optimized thermal profile of PCR was initial denaturation at 94°C for 4 min, followed by 39 cycles of denaturation at 94°C for 40 s, annealing at 65°C for 45 s and extension at 72°C for 40 s, and a final extension of 72°C for 7 min. The amplifications were confirmed on 1.2 percent agarose gel in 1X TAE buffer with a distinct 600 bp band, run parallel to standard DNA molecular weight marker (100 bp DNA ladder), and visualized under Geldoc system (FireReader V10, Uvitec Cambridge, United Kingdom).

#### 2.3.4 ITS rDNA region and GAPDH amplification

For the ITS region amplification, primers were synthesized (ITS1: 5′-TCCTCCGCTTATTGATATGC-3′ and ITS4: 5′-GAAGTAAAAGTCGTAACAAGG-3′) from Agrigenome Labs Pvt. Ltd. (India) as described by White et al. (1990), and PCR was run with the following conditions: initial denaturation at 95°C for 4 min, followed by 35 cycles of denaturation at 95°C for 45 s, annealing at 55°C for 45 s, extension at 72°C for 1 min, and a final extension for 10 min at 72°C. For GAPDH gene amplification, primers were synthesized (gpd1: 5′-ATACACTGCCACCCAGAAGG-3′ and gpd2: 5′-TCGATGCGAACAGTCAAGTC-3′) as described by Manamgoda et al. (2012) and PCR was run with the following conditions: initial denaturation at 96°C for 2 min, followed by 35 cycles of denaturation at 96°C for 1 min, annealing at 52°C for 1 min, extension at 72°C for 45 s, and a final extension at 72°C for 10 min. The amplified PCR product of ITS and GAPDH was visualized on 1.2 percent (w/v) agarose gel, and the expected band was purified from the gel using a Wizard® SV gel and PCR clean-up system (Promega, United Kingdom) and sent for sequencing by Sanger dideoxy sequencing method (Barcode Biosciences Pvt. Ltd., Bengaluru). The resultant sequences were deposited to GenBank of the NCBI database.

#### 2.3.5 ITS rDNA region and GAPDH phylogenetic analyzes

The ITS and GAPDH sequences of all fungal isolates were used as query sequences against the NCBI database^4^ using the basic local alignment search tool (BLAST) to reveal their relationships to published sequences. All isolates were subjected to an evolutionary analysis with other closely related organisms’ sequences retrieved from NCBI as references. All the sequences were aligned, and a phylogeny tree was created using the maximum likelihood method with 1,000 bootstrap replications as a test of phylogeny using MEGA version 11 (Tamura et al., 2021).^5^

### 2.4 Pathogenicity assessment

For all the isolates obtained, a pathogenicity test was conducted during the *Rabi* season of 2021–2022 under polyhouse conditions using the susceptible wheat variety ‘Sonalika’ and the moderately resistant variety “HD2733” (Verma et al., 2020). Using both a moderately resistant and susceptible variety for pathogenicity assessment aligns with the traits observed in commercially grown cultivars, which display a spectrum from moderately resistant to susceptible (Roy et al., 2023). The dual variety approach precisely captures the common dynamics of commercial wheat cultivation, ensuring the practical relevance of our study. To prepare the inoculum, all isolates were mass multiplied on 100 g of sterilized sorghum grains by inoculating them with fungal discs under aseptic conditions. These flasks were then kept in the dark at 25°C for 20–30 days and mixed well at regular intervals to ensure homogenous growth. After all the grains were completely coated with fungal spores, inoculum was harvested by mixing the grains with 200 mL of double distilled water (DDW). They were mixed thoroughly using a magnetic shaker (Tarsons digital spinot, Kolkata, India) and sieved through three layers of muslin cloth. The spore concentration was adjusted to 10^4^ spores/ml by adding double distilled water to each sample. A few drops of polyoxyethylene-20-sorbitan monolaurate (Tween 20) were added to each spore suspension as a spreader and sticker to facilitate adherence and even distribution of inoculum on seedling leaves (Verma et al., 2020).

The pot experiment was conducted in the polyhouse facility of the Division of Plant Pathology, IARI, New Delhi. Wheat seeds (Sonalika and HD2733) were sown in 4-inch plastic pots with a soil mixture of FYM, sand, and field soil (1,1,2); 10–15 seeds per pot were sown, and three pots per isolate were maintained. Nitrogen-based fertilizer was added to each pot to promote seedling growth at the time of the first irrigation. Two-week-old seedlings were inoculated at the 2–3 leaf stage [GS 13 of Zadoks et al. (1974)] using a hand atomizer. The spore suspension was sprayed sufficiently till runoff. Inoculated plants were put in chambers and incubated in darkness for 18 h at 20–22°C with 100 percent humidity. After that, the plants were grown in growth cabinets with a 12-h photoperiod (Iftikhar et al., 2008). Infected leaves after 4–5 dpi were evaluated for the number of lesions, the size of the lesions, and the average disease index (ADI) using a 0–5 scale developed by Adlakha et al. (1984). All the isolates were tested at least twice for pathogenicity.

### 2.5 Population dynamics in soil

Another polyhouse experiment was conducted in order to assess the impact of two different varieties (Sonalika and HD2733) on the survival and multiplication of fungal isolates in the rhizosphere. For this, fungal isolates, mass multiplied on sorghum grains, were inoculated in field soil taken in pots. In order to provide the mass-multiplied fungal cultures adequate time for development and colonization, they were inoculated (@10 g/kg soil) into the soil 2 days prior to sowing. After every 15 days (15, 30, 45, 60, and 75 days following planting), soil samples were drawn from the rhizosphere of both varieties and kept at −80°C for further processing. Every isolate was inoculated on a total of 6 pots (3/variety) and as a control, pots filled with soil without fungal inoculation were used.

#### 2.5.1 Serial dilution or colony count assay

The soil samples drawn at periodic intervals were serially diluted (10^−3^) and plated on PDA media using L-shaped spreaders. These plates were then incubated for 7 days at 28°C. Colonies on the plates were counted on the seventh day, and observations were recorded. The concentration of fungal population in the soil during the cropping season was later determined using this data.

#### 2.5.2 Absolute quantification by real-time assay

##### 2.5.2.1 Soil DNA extraction and quantification

The DNA from soil samples was extracted using ZR Soil Microbe DNA KitTM (Cat. no. D6001; Zymo Research Corp., United States). Extraction was performed using the manufacturer’s instructions. The purity and quantity of soil DNA were checked using a Nanodrop spectrophotometer (Thermo Fisher Scientific Inc. 2000, United States) and kept at −20°C until further processing.

##### 2.5.2.2 Real-time PCR assays

All qPCR assays involving 32 isolates each at 5 different time points were performed on the BioRad CFX96 system (Bio-Rad Laboratories, Inc. India) in the genomics laboratory of Discovery Centre, ICAR-IARI, New Delhi, India. Specific qPCR-based primers were designed from PCR-based SCAR_600_ primers used earlier in this study (Aggarwal et al., 2011) to selectively amplify the target DNA from the total soil DNA in order to quantify our isolates in soil samples. The total reaction volumes were 20 μL which consists of DyNAmo ColorFlash SYBR Green qPCR master mix (Thermo Fisher Scientific, Massachusetts, USA), DEPC-treated water (SRL Pvt. Ltd., Mumbai, India), and 200 nM of both forward and reverse primers. Template DNA were added in 1 μL volume per reaction. All the samples were performed with 3 technical replicates including triplicates of no-template controls, containing DEPC-treated water in each run. The optimized qPCR profile was initial denaturation at 95°C for 5 min, denaturation at 95°C for 30 s, annealing at 60°C for 45 s, and final extension at 72°C for 1 min with 40 cycles. After each qPCR run, melting curve analysis was performed to ensure the presence of the desired amplicon.

##### 2.5.2.3 Quantification by standard curve (SC) method

The SC method involves a dilution series of defined target DNA concentration (standards of target DNA: 100 ng, 10 ng, 1 ng, 0.1 ng, 0.01 ng, 0.001 ng, 0.0001 ng). The linear regression of log (*N*_0 standard_) versus *C_T_* gives the constants c and slope m of the standard curve (Supplementary Figure S2). The target DNA copy number in the soil samples, *N*_0 sample_, can be calculated based on the regression equation of the standard curve. Genomic DNA was used to prepare a standard curve (Brankatschk et al., 2012).


$$C_{T\mathrm{sample}}=a+b\times \log\left(N_{0\;\mathrm{sample}}\right)$$

The target DNA copy number in soil samples (*c*_target_ [copies μl^−1^]) was calculated from the total DNA concentration (*c*_DNA_ [ng μl^−1^]), the length of target DNA fragment (*l*_DNA_ [bp]), number of targets per DNA fragment (*n*_target_[copies]), the Avogadro constant (*N_A_*) (6.022 × 10^23^ bp mol^−1^), and the average weight of a double-stranded base pair (*M_bp_*) (660 g mol^−1^ = 6.6 × 10^11^ ng mol^−1^) (Brankatschk et al., 2012).


$$c_{\mathrm{target}}=n_{\mathrm{target}}\times \frac{c_{\mathrm{DNA}}\times N_{A}}{l_{\mathrm{DNA}}\times M_{\mathrm{bp}}}$$

### 2.6 Statistical analyzes

All the statistical analysis was performed by using IBM SPSS Statistics version 20 software for analysis of variance. Visual representation of the data and results were made using R statistical software (v4.2.3; R Core Team, 2021).^6^

## 3 Results

### 3.1 Morphological characterization

**Figure 1:**
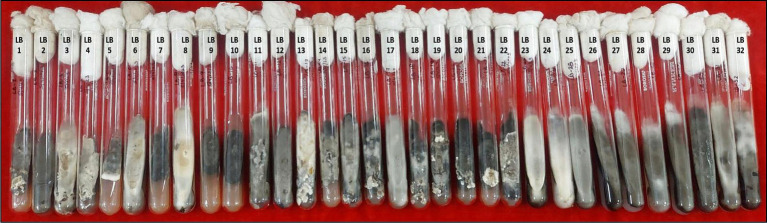
Purified mono-conidial cultures of 32 leaf blight/spot blotch isolates on potato dextrose agar slants (15 days old).

A total of 32 putative isolates of *Bipolaris* were established from 10 different states–representing all wheat growing zones in India–exhibiting similar symptoms (Table 1). Based on the colony morphology, these isolates were categorized into five classes: (I) black suppressed growth; (II) brown/dull black suppressed growth; (III) gray with white patches cottony growth; (IV) dull white and/or greenish black fluffy growth; and (V) white fluffy growth, based on colony color and development pattern (Supplementary Figure S3). The population under study revealed a high frequency of the gray with white spots colony type (31.25%) and a low frequency of the white fluffy type (12.5%) (Figure 1; Table 1).

**Table 1:** List of 32 leaf blight/spot blotch-causing isolates collected from six wheat growing zones of India.

S. no	Wheat growing zones	Place of collection	Isolate name	Species	Source of isolation	Colony type
1	North Hill zone (NH)	Uttarakhand	LB-30	*Exserohilum rostratum*	Black point seed	Brown /dull black suppressed growth
2	North Hill zone (NH)	Uttarakhand	LB-31	*Exserohilum rostratum*	Black point seed	Dull white/greenish black fluffy growth
3	North Western Plain Zone (NWPZ)	New Delhi	LB-5	*Bipolaris sorokiniana*	Infected leaf	Dull white/greenish black fluffy growth
4	North Western Plain Zone (NWPZ)	Haryana	LB-6	*Bipolaris sorokiniana*	Infected leaf	White fluffy growth
5	North Western Plain Zone (NWPZ)	New Delhi	LB-8	*Bipolaris sorokiniana*	Infected leaf	Dull white/greenish black fluffy growth
6	North Western Plain Zone (NWPZ)	New Delhi	LB-11	*Bipolaris sorokiniana*	Infected leaf	Brown /dull black suppressed growth
7	North Western Plain Zone (NWPZ)	New Delhi	LB-12	*Bipolaris sorokiniana*	Infected leaf	Brown /dull black suppressed growth
8	North Western Plain Zone (NWPZ)	New Delhi	LB-13	*Bipolaris sorokiniana*	Infected leaf	Dull white/greenish black fluffy growth
9	North Western Plain Zone (NWPZ)	New Delhi	LB-17	*Bipolaris sorokiniana*	Infected leaf	White fluffy growth
10	North Western Plain Zone (NWPZ)	Rajasthan	LB-19	*Bipolaris sorokiniana*	Infected leaf	Black suppressed growth
11	North Western Plain Zone (NWPZ)	Rajasthan	LB-20	*Bipolaris sorokiniana*	Infected leaf	Black suppressed growth
12	North Western Plain Zone (NWPZ)	Rajasthan	LB-21	*Bipolaris sorokiniana*	Infected leaf	Black suppressed growth
13	North Western Plain Zone (NWPZ)	Rajasthan	LB-22	*Bipolaris sorokiniana*	Infected leaf	Dull white/greenish black fluffy growth
14	North Western Plain Zone (NWPZ)	New Delhi	LB-28	*Bipolaris spicifera*	Infected leaf	Dull white/greenish black fluffy growth
15	North Western Plain Zone (NWPZ)	New Delhi	LB-29	*Bipolaris spicifera*	Infected leaf	Dull white/greenish black fluffy growth
16	North Western Plain Zone (NWPZ)	Haryana	LB-32	*Exserohilum rostratum*	Infected leaf	Brown /dull black suppressed growth
17	North Eastern Plain Zone (NEPZ)	Uttar Pradesh	LB-1	*Bipolaris sorokiniana*	Infected leaf	Dull white/greenish black fluffy growth
18	North Eastern Plain Zone (NEPZ)	Uttar Pradesh	LB-4	*Bipolaris sorokiniana*	Infected leaf	Dull white/greenish black fluffy growth
19	North Eastern Plain Zone (NEPZ)	Bihar	LB-14	*Bipolaris sorokiniana*	Infected leaf	Dull white/greenish black fluffy growth
20	North Eastern Plain Zone (NEPZ)	Bihar	LB-15	*Bipolaris sorokiniana*	Infected leaf	Dull white/greenish black fluffy growth
21	North Eastern Plain Zone (NEPZ)	Bihar	LB-16	*Bipolaris sorokiniana*	Infected leaf	Dull white/greenish black fluffy growth
22	North Eastern Plain Zone (NEPZ)	Bihar	LB-18	*Bipolaris sorokiniana*	Infected leaf	Black suppressed growth
23	North Eastern Plain Zone (NEPZ)	Uttar Pradesh	LB-24	*Bipolaris sorokiniana*	Infected leaf	White fluffy growth
24	Central Zone (CZ)	Chhattisgarh	LB-25	*Bipolaris oryzae*	Infected leaf	Dull white/greenish black fluffy growth
25	Peninsular Zone (PZ)	Maharashtra	LB-2	*Bipolaris sorokiniana*	Infected leaf	Dull white/greenish black fluffy growth
26	Peninsular Zone (PZ)	Karnataka	LB-23	*Bipolaris sorokiniana*	Infected leaf	White fluffy growth
27	Southern Hills Zone (SHZ)	Tamil Nadu	LB-3	*Bipolaris sorokiniana*	Infected leaf	Brown /dull black suppressed growth
28	Southern Hills Zone (SHZ)	Tamil Nadu	LB-7	*Bipolaris sorokiniana*	Infected leaf	Black suppressed growth
29	Southern Hills Zone (SHZ)	Tamil Nadu	LB-9	*Bipolaris sorokiniana*	Infected leaf	Brown /dull black suppressed growth
30	Southern Hills Zone (SHZ)	Tamil Nadu	LB-10	*Bipolaris sorokiniana*	Infected leaf	Black suppressed growth
31	Southern Hills Zone (SHZ)	Tamil Nadu	LB-26	*Bipolaris spicifera*	Infected leaf	Dull white/greenish black fluffy growth
32	Southern Hills Zone (SHZ)	Tamil Nadu	LB-27	*Bipolaris spicifera*	Infected leaf	Dull white/greenish black fluffy growth

The isolate details including the assigned name, their place of collection, source of isolation, identified species, and their colony type are provided.

**Figure 2:**
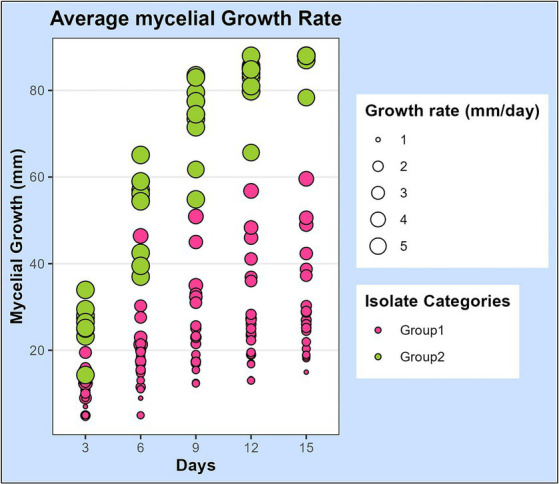
Scatterplot representing the mycelial growth rate of 32 leaf blight/spot blotch isolates on potato dextrose agar medium over a period of 15 days. Dots represent 32 isolates at 3, 6, 9, 12, and 15 days after plating on PDA medium. Size of the dots indicates their average growth rate/day (mm/day). Color of dots represents the two categories of isolates: “Group 1” isolates are slow-growing with a lesser average growth rate (<5 mm/day) and “Group 2” isolates are fast-growing with a higher average growth rate (>5 mm/day).

The mycelial growth of all isolates was observed 3, 6, 9, 12, and 15 days after inoculation (DAI) on PDA plates and the average growth rate per day was calculated. The growth rates of isolates were statistically analyzed and observed to differ significantly among the isolates (Supplementary Table S1). Additionally, we also observed that the growth rate of a single isolate fluctuated at different growth intervals. The development patterns of isolates also differed, with some growing at a faster rate initially and others at a later stage. Based on the growth pattern of isolates, it was noticed that there were two extremes: one set of isolates with a growth rate greater than 5 mm/day and the other with a growth rate considerably lower than that, between 1 and 3 mm/day. Based on this information, the isolates were categorized into two groups (Figure 2).

For spore morphology, microscopic observations were taken in 10-day-old cultures using a phase contrast microscope (Nikon Eclipse C*i*) at 10X and 40X resolution (Table 2). All the isolates except LB-23, LB-25, and LB-24 were able to produce conidia on PDA medium (Supplementary Figure S4). Average conidial length ranged from 21.89 μm (LB-26) to 98.47 μm (LB-13) and average conidial width ranged from 8.77 μm (LB-27) to 25.54 μm (LB-14). The dimensions varied significantly among the isolates. The sporulation capability of 32 isolates ranged from zero to 8.57 × 10^7^ spores/ml. The isolate LB-28 from New Delhi had the highest sporulation (8.57 × 10^7^ spores/ml), whereas, isolate LB-6 from Karnal, Haryana, had the lowest sporulation (0.27 × 10^7^ spores/ml). Additionally, the number of septa varied significantly amongst the isolates, with isolate LB-31 having the highest number of septa (14.61) and isolates LB-26, LB-27, LB-28, and LB-29 having the least number of septa (exactly 3 in all the spores) (Table 2).

**Table 2:** Measurement of spore morphological parameters: sporulation, spore size, and septation of 32 leaf blight/spot blotch isolates taken for the study.

S. no	Isolate	Length	Width	Sporulation	Septation
1	LB-1	68.69	19.85	5.20	7.23
2	LB-2	74.69	21.81	4.00	6.91
3	LB-3	84.33	24.02	2.20	8.22
4	LB-4	95.18	20.88	7.13	7.51
5	LB-5	62.03	21.51	0.43	6.23
6	LB-6	76.83	23.05	0.27	5.31
7	LB-7	56.13	21.86	5.20	6.63
8	LB-8	71.45	25.25	4.13	7.95
9	LB-9	82.26	24.47	4.50	5.11
10	LB-10	75.22	24.91	5.60	8.22
11	LB-11	62.43	21.77	1.57	5.89
12	LB-12	53.46	22.46	1.87	5.01
13	LB-13	98.47	23.90	7.80	8.53
14	LB-14	91.48	25.54	8.47	8.72
15	LB-15	64.49	24.16	8.33	9.12
16	LB-16	61.56	21.53	8.03	8.86
17	LB-17	70.06	21.12	1.33	6.54
18	LB-18	75.16	22.39	7.70	8.72
19	LB-19	55.31	21.37	7.83	7.15
20	LB-20	54.14	19.63	8.17	6.22
21	LB-21	60.74	20.18	8.37	7.19
22	LB-22	59.35	20.51	7.73	8.90
23	LB-23	–	–	–	–
24	LB-24	–	–	–	–
25	LB-25	–	–	–	–
26	LB-26	21.89	9.14	8.43	3.00
27	LB-27	22.95	8.77	8.40	3.00
28	LB-28	23.96	10.85	8.57	3.00
29	LB-29	23.49	11.84	8.30	3.00
30	LB-30	83.55	17.25	6.63	13.55
31	LB-31	92.49	16.50	8.07	14.61
32	LB-32	95.31	15.67	8.17	12.98
C.D. (*α* = 0.05)		2.70	1.40	0.18	
SE(m)		0.95	0.49	0.06	
SE(d)		1.34	0.70	0.09	
C.V.		2.51	4.25	2.04	

^*^Isolates LB-23, LB-24, and LB-25 are sterile cultures.

Microscopic observations provided critical information regarding isolate identification. Group I isolates with lower growth rates in the culture medium were identified as *B. sorokiniana* isolates, whereas, Group II isolates with higher growth rates were further subdivided based on their spore structures into two classes: *B. spicifera* isolates and *Exserohilum rostratum* isolates. It must be noted that isolates LB-23, LB-24, and LB-25 were not designated to any of the above-mentioned groups (non-sporulating) because they were sterile cultures and their spores could not be examined (Table 2).

### 3.2 Molecular characterization

All pathogenic isolates employed in the present study were first identified based on cultural and morphological characteristics and further verified through molecular identification. To our surprise, the PCR-based diagnostic SCAR marker was able to produce a visible and distinct 600 bp unique band in all the isolates taken for the study irrespective of their morphological identities (Supplementary Figure S5). Hence, in order to evaluate the marker for its diagnostic ability, various other plant pathogenic and non-pathogenic fungi were taken as negative controls, and in all the cases, the marker did not produce any band. PCR amplification with the SCAR marker was repeated with newly synthesized primers as well to minimize handling errors, but the results were repeatable.

The ITS and GAPDH region was amplified successfully by fungal-specific universal pairs of primers (ITS1-F and ITS4-R; gpd1-F and gpd-R) and sequenced for all the isolates taken in the study. The resulting sequences were first trimmed for high-quality bases and later aligned and assembled with BioEdit sequence alignment editor version 7.2.5.0 for the generation of consensus sequences (Hall, 1999). Further, these rDNA and GAPDH sequences were analyzed using NCBI-BLAST.

The BLAST analysis of ITS rDNA sequences corroborates with the morphological findings by confirming the presence of three groups of pathogens: *B. sorokiniana*, *B. spicifera*, and *E. rostratum*. All the sequences shared 99–100 percent similarity with 100 percent query cover with one of the pathogenic groups identified through morphological studies. The BLAST analysis also revealed the presence of a fourth group, i.e., *B. oryzae*, represented by a single isolate LB-25 (Table 1). Since isolate LB-25 was among the non-sporulating isolates, its identity was only revealed through similarity searches using the BLASTX tool against the NCBI database. The ITS rDNA sequences of the 32 isolates used in the study have been deposited in the NCBI GenBank database with accession numbers OQ845799-OQ845830 (Supplementary Table S2).

**Figure 3:**
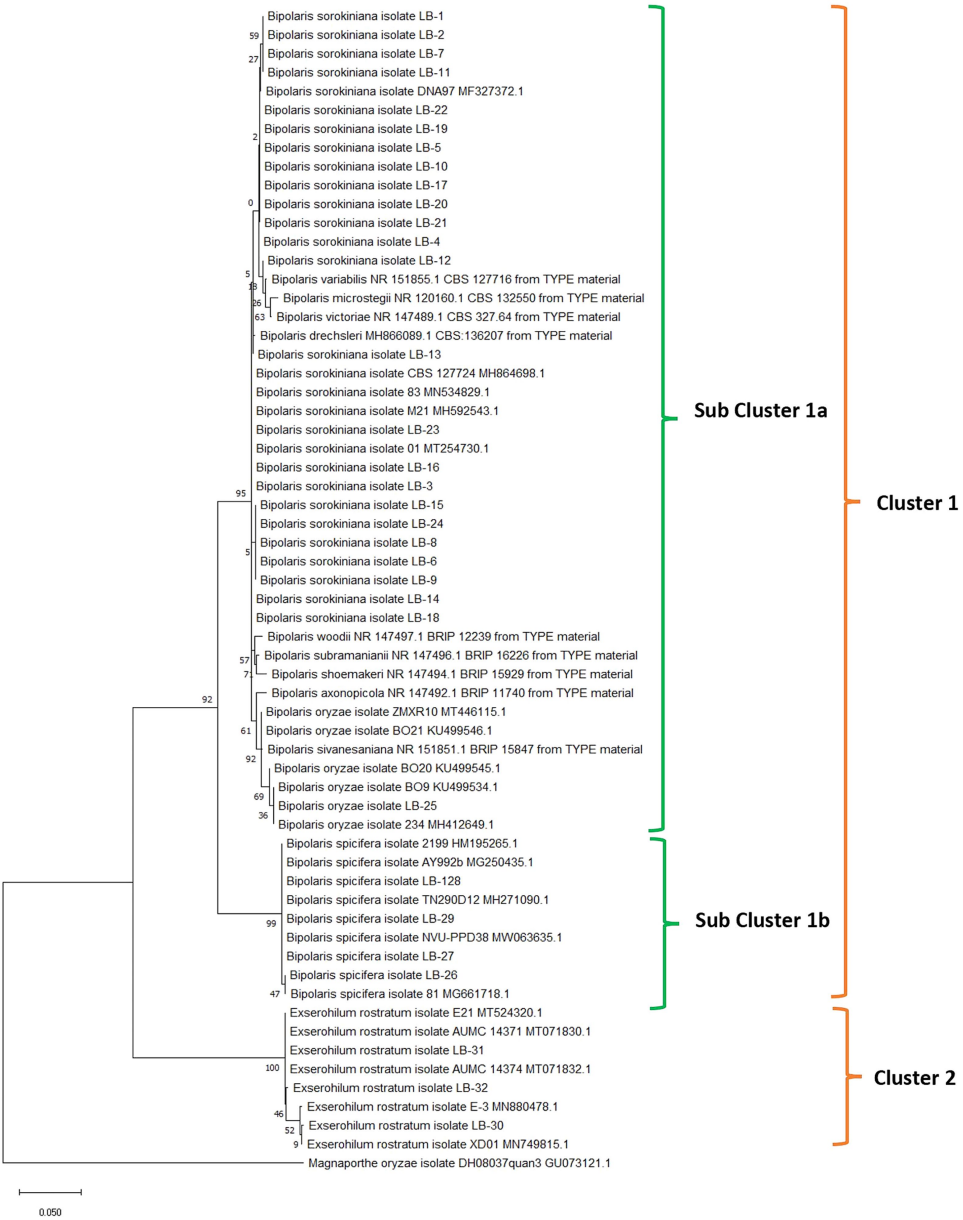
Phylogenetic analysis of rDNA-ITS region of fungal isolates obtained from leaf blight-infected wheat leaves and seeds in wheat growing zones of India. The evolutionary analysis of *Bipolaris sorokiniana*, *Bipolaris spicifera*, *Bipolaris oryzae,* and *Exserohilum rostratum* is inferred based on rDNA-ITS sequences using the maximum likelihood method with 1,000 replications. Reference sequences (5 for each cluster) retrieved from NCBI are provided with accession numbers along with species names. The rDNA-ITS sequence of *Magnaporthe oryzae* was taken as an outgroup.

Nucleotide sequences of ITS locus were aligned with the program Clustal W (Thompson et al., 1994), and manually optimized using the program MEGA11 (Tamura et al., 2021). The alignments were analyzed using the maximum likelihood method and the Tamura-Nei model with 1,000 replications (Tamura and Nei, 1993). The phylogenetic tree was inferred with all 32 isolates used in the study, 20 reference sequences (5 sequences for each pathogenic group), and sequences of ex-type cultures retrieved from the NCBI database. The *Magnaporthe oryzae* isolate DH08037quan3 ITS sequence was used as an outgroup. The phylogenetic tree clearly clustered into two major groups. Isolates of *E. rostratum* distinctly form a separate cluster (Cluster 2) away from the Bipolaris group of pathogens (Cluster 1). Cluster 1 further bifurcates to form two subclusters. Subcluster 1a consists of *B. sorokiniana* and *B. oryzae* and subcluster 1b consists of *B. spicifera* (Figure 3).

**Figure 4:**
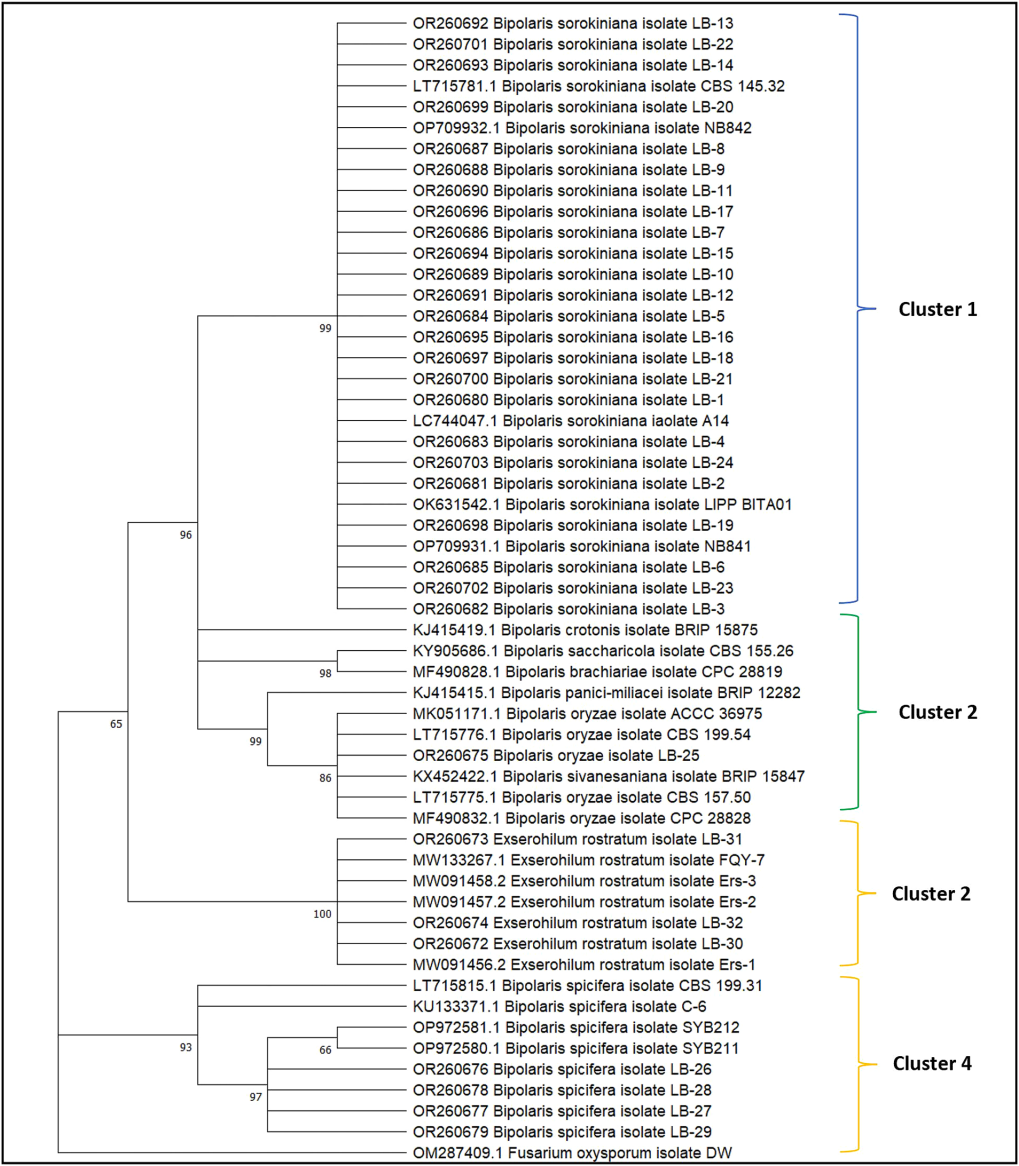
Phylogenetic analysis of the GAPDH gene of 32 fungal isolates obtained from leaf blight-infected wheat leaves and seeds in wheat growing zones of India. The evolutionary analysis of *Bipolaris sorokiniana*, *Bipolaris spicifera*, *Bipolaris oryzae,* and *Exserohilum rostratum* is inferred based on GAPDH sequences using the maximum likelihood method with 1,000 replications. Reference sequences along with sequences of ex-type species retrieved from NCBI are provided with accession numbers. The GAPDH sequence of *Fusarium oxysporum* was taken as an outgroup.

The BLAST analysis of GAPDH sequences validates the ITS results and establishes the identity of all isolates with 99–100 percent similarity and query cover. All GAPDH sequences generated in the present study have been deposited in the NCBI GenBank database with accession numbers OR260672-OR260703 (Figure 4; Supplementary Table S2). The phylogenetic tree was generated with 32 GAPDH sequences of all isolates, 15 reference sequences, and sequences of ex-type cultures retrieved from the NCBI database. All the sequences were aligned with the program Clustal W (Thompson et al., 1994), and manually optimized using the program MEGA11 (Tamura et al., 2021). The alignments were analyzed using the maximum likelihood method and the Tamura-Nei model with 1,000 replications (Tamura and Nei, 1993). All isolates with similar identities were clustered together in a single clade. *Fusarium oxysporum* isolate DW (China) GAPDH sequence was used as an outgroup (Figure 4).

A concatenated phylogenetic tree with ITS and GAPDH was also constructed using maximum likelihood criteria through RAxML version 8.0.0 (Stamatakis, 2014) under a GTRGAMMA model with 1,000 replications provided in Supplementary Figure S6.

### 3.3 Pathogenicity test

**Figure 5:**
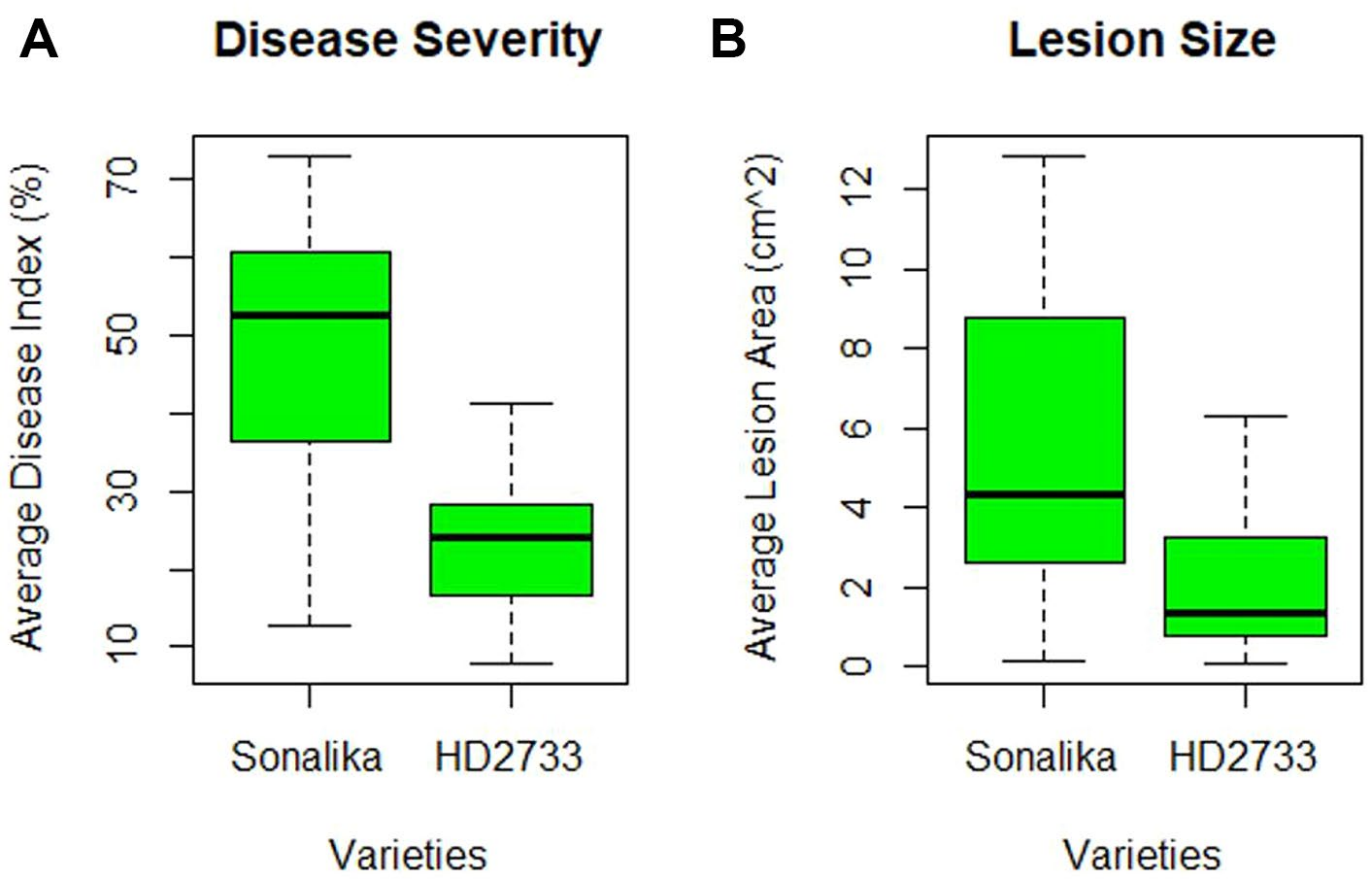
Varietal difference between two wheat varieties, HD2733 (moderately resistant) and Sonalika (susceptible), on the basis of **(A)** disease severity (in percentage), and **(B)** lesion size (in cm^2^) produced by 32 leaf blight/spot blotch isolates taken for the study.

**Figure 6:**
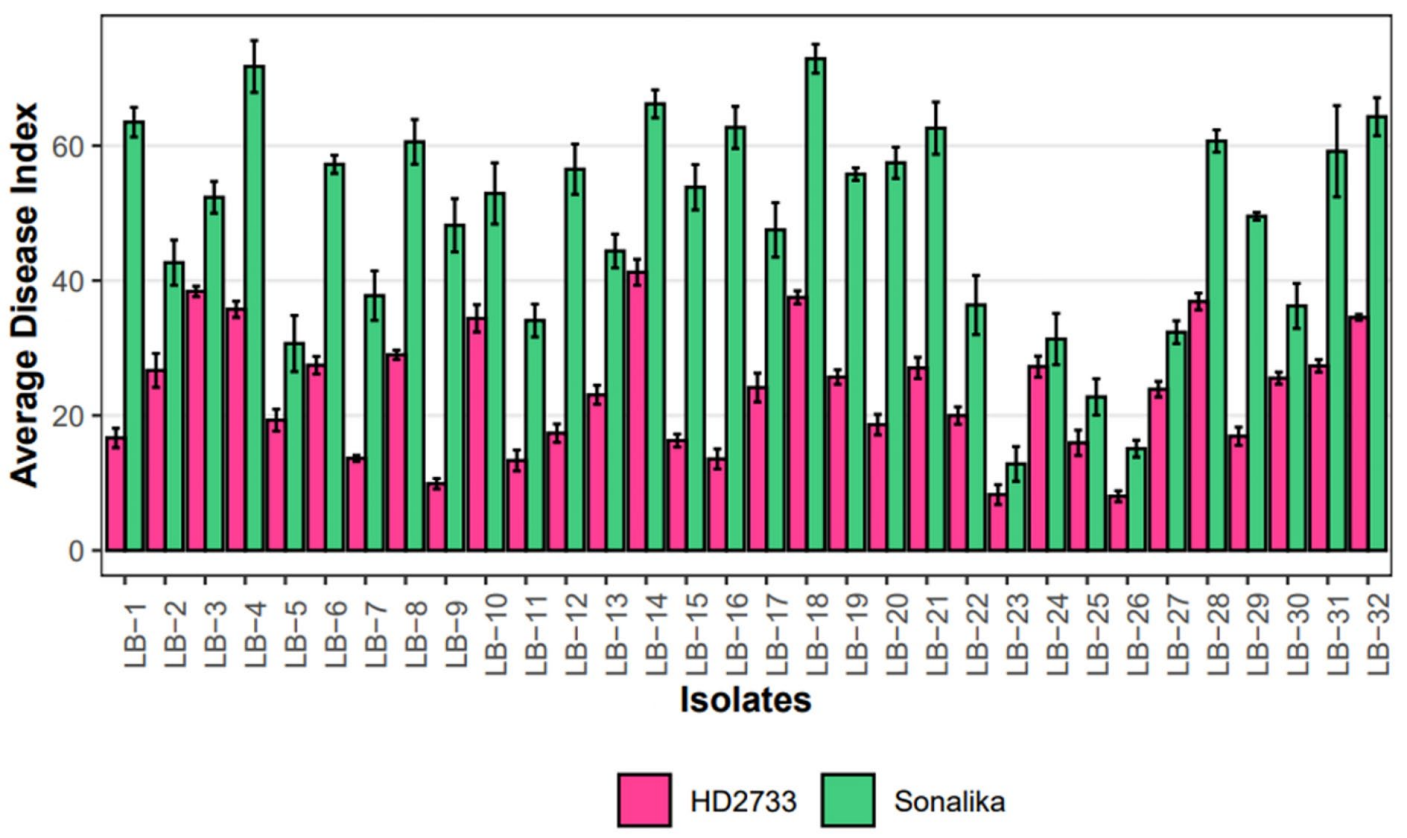
Grouped Barplot representing disease severity, measured as average disease index (ADI), of 32 leaf blight isolates on Sonalika (susceptible variety) and HD2733 (moderately resistant variety). Bars represent standard deviation of the mean.

A pathogenicity test conducted on all 32 isolates confirmed that each isolate employed in this study is a causal agent of leaf blight/spot blotch on an individual basis (Supplementary Figure S7). The experiment was conducted in the polyhouse facility of the Division of Plant Pathology, IARI, New Delhi, India. Two wheat varieties, Sonalika (a susceptible variety) and HD2733 (a moderately resistant variety) (Figure 5), were artificially inoculated and incubated in the dark for 18 h at 20–22°C with >90 percent relative humidity, followed by a 12-h alternate photoperiod in growth cabinets. After 6–7 days of inoculation, brown necrotic spots started appearing, accompanied by a yellow halo, which were scored, and the infection index was determined (Adlakha et al., 1984). All the isolates were pathogenic to varying extents, and their association was confirmed by proving the Koch Postulates (Figure 6).

The disease severity of *B. sorokiniana* isolates ranged from 72.89 (LB-18) to 12.82 (LB-23) on Sonalika and 41.23 (LB-14) to 8.27 (LB-23) on HD2733. Also, the average lesion area ranged from 12.83 mm^2^ (LB-18) to 0.16 mm^2^ (LB-23) on Sonalika and from 5.42 mm^2^ (LB-18) to 0.11 mm^2^ (LB-23) on HD2733. The disease severity of *B. spicifera* isolates ranged from 60.68 (LB-28) to 15.08 (LB-26) on Sonalika and from 36.90 (LB-28) to 8.03 (LB-26) on HD2733. Also, the average lesion area ranged from 4.43 mm^2^ (LB-27) to 0.34 mm^2^ (LB-28) on Sonalika and from 1.32 mm^2^ (LB-29) to 0.12 mm^2^ (LB-26) on HD2733. The disease severity of *E. rostratum* isolates ranged from 64.29 (LB-32) to 36.23 (LB-30) on Sonalika and from 34.55 (LB-32) to 25.54 (LB-30) on HD2733. Also, the average lesion area ranged from 11.07 mm^2^ (LB-30) to 5.59 mm^2^ (LB-31) on Sonalika and from 6.30 mm^2^ (LB-32) to 1.36 mm^2^ (LB-31) on HD2733. The disease severity of *B. oryzae* isolate (LB-25) was 22.75 on Sonalika and 15.95 on HD2733, with an average lesion area of 1.43 mm^2^ on Sonalika and 0.53 mm^2^ on HD2733 (Figure 5; Table 3).

**Table 3:** Pathogenicity assessment of 32 leaf blight/spot blotch isolates on wheat varieties Sonalika (susceptible) and HD2733 under polyhouse conditions from 2020–2022.

S.No	Isolate	Average disease index (ADI) (%)		Lesion size (in cm)					
				Sonalika			HD2733		
		Sonalika	HD2733	Length	Width	Area	Length	Width	Area
1	LB-1	63.48	16.67	5.64	1.69	9.53	2.15	1.58	3.40
2	LB-2	42.66	26.69	2.38	1.73	4.12	1.26	0.57	0.72
3	LB-3	52.33	38.39	3.94	1.74	6.86	2.54	1.04	2.64
4	LB-4	71.73	35.74	4.28	2.12	9.07	2.93	1.45	4.25
5	LB-5	30.67	19.31	2.62	1.43	3.76	1.74	1.14	1.99
6	LB-6	57.23	27.45	2.46	1.68	4.13	1.31	0.63	0.82
7	LB-7	37.77	13.64	2.16	1.31	2.83	1.35	0.72	0.97
8	LB-8	60.55	29.01	3.84	1.46	5.59	2.14	1.17	2.50
9	LB-9	48.19	9.89	0.79	0.34	0.27	0.53	0.36	0.19
10	LB-10	52.91	34.39	1.76	1.43	2.52	1.28	0.91	1.17
11	LB-11	34.08	13.33	3.19	1.11	3.53	0.53	0.36	0.19
12	LB-12	56.50	17.38	1.87	0.98	1.84	1.30	0.54	0.70
13	LB-13	44.38	23.06	2.90	1.35	3.91	1.43	0.64	0.92
14	LB-14	66.19	41.23	6.47	1.75	11.33	3.79	1.31	4.96
15	LB-15	53.83	16.29	5.15	1.62	8.33	2.37	1.37	3.25
16	LB-16	62.70	13.55	4.58	2.12	9.71	2.15	1.54	3.32
17	LB-17	47.52	24.14	1.85	1.06	1.96	1.65	0.75	1.24
18	LB-18	72.89	37.51	5.13	2.50	12.83	3.23	1.68	5.42
19	LB-19	55.77	25.70	3.11	1.56	4.87	1.23	0.64	0.79
20	LB-20	57.46	18.65	3.70	2.31	8.54	2.00	1.75	3.50
21	LB-21	62.59	27.05	5.09	2.06	10.50	3.49	1.57	5.50
22	LB-22	36.38	19.99	2.99	1.67	4.98	1.73	1.10	1.91
23	LB-23	12.82	8.27	0.63	0.25	0.16	0.44	0.26	0.11
24	LB-24	31.33	27.24	2.62	1.61	4.22	1.54	0.94	1.45
25	LB-25	22.75	15.95	1.34	1.07	1.43	0.96	0.55	0.53
26	LB-26	15.08	8.03	0.73	0.51	0.37	0.51	0.23	0.12
27	LB-27	32.34	23.89	3.69	1.20	4.43	1.28	0.84	1.08
28	LB-28	60.68	36.90	0.82	0.46	0.37	0.53	0.26	0.14
29	LB-29	49.52	16.93	2.14	1.25	2.68	1.24	1.06	1.32
30	LB-30	36.23	25.54	4.90	2.26	11.07	1.76	1.51	2.65
31	LB-31	59.16	27.36	3.27	1.71	5.59	1.77	0.77	1.36
32	LB-32	64.29	34.55	5.67	1.69	9.58	3.99	1.58	6.30
C.D. (*α* = 0.05)		5.24	2.27	0.30	0.05		0.09	0.05	
SE(m)		1.85	0.80	0.10	0.02		0.03	0.01	
SE(d)		2.62	1.13	0.15	0.02		0.04	0.02	
C.V.		6.61	5.89	5.88	2.32		3.39	3.15	

### 3.4 Soil population dynamics

#### 3.4.1 Soil inoculum quantification via serial dilution and colony count method

Plates were observed after 4 days of plating and colonies resembling the pathogens discussed above were counted and documented. On variety Sonalika, isolate LB-27 reported the maximum (30.1 × 10^3^) and isolate LB-4 recorded the least (2.6 × 10^3^) average colony forming units per gram of soil, whereas on HD2733, isolate LB-7 measured the highest (15.9 × 10^3^) and isolate LB-4 measured the least (1.4 × 10^3^) colony forming units per gram of soil. The average inoculum load was much lower in HD2733 compared to Sonalika for all isolates and at all time points except for isolates LB-3 and LB-8 (Supplementary Tables S3a,b).

#### 3.4.2 Soil inoculum quantification By qPCR

**Figure 7:**
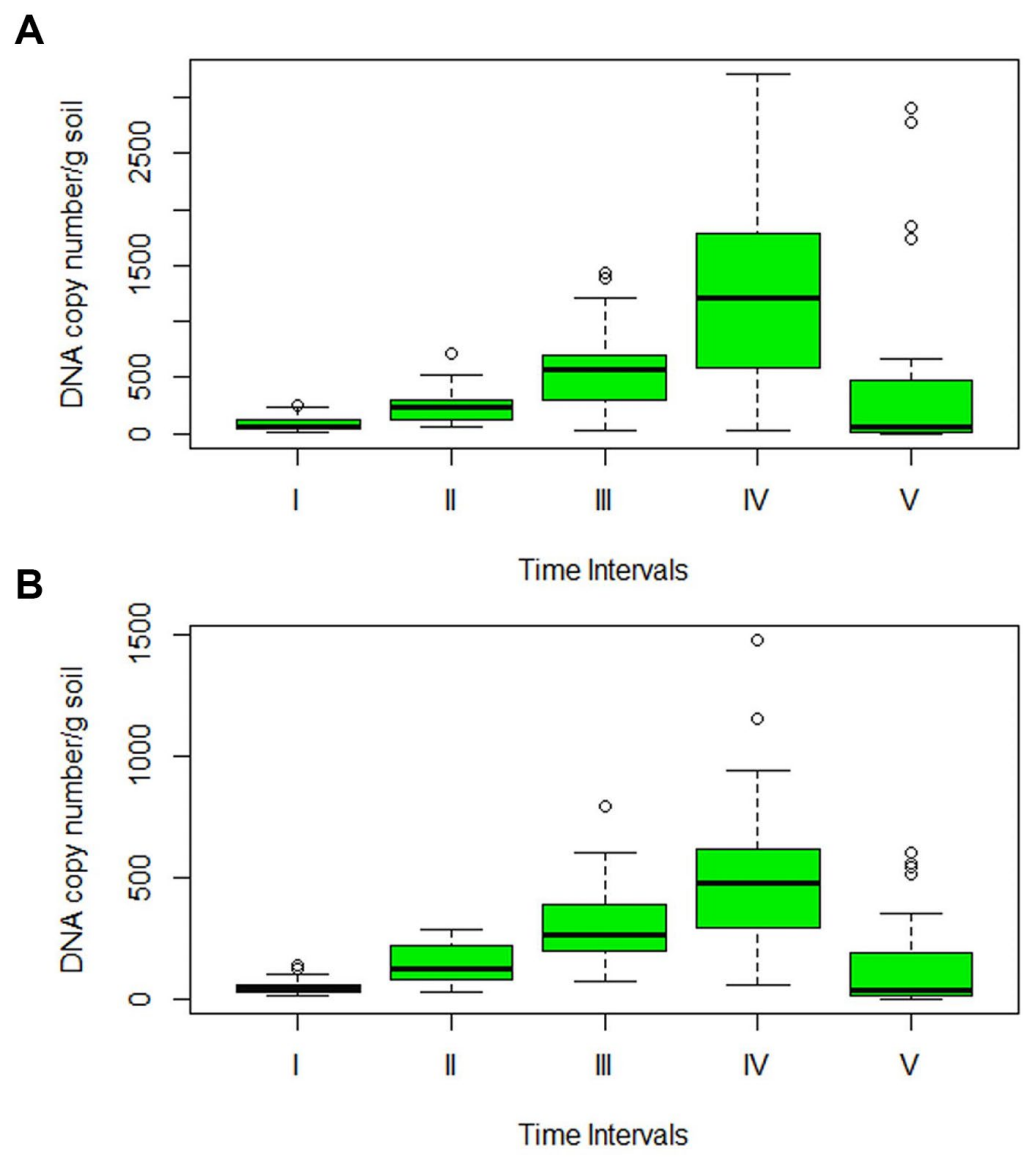
Box plot of soil population dynamics studies of 32 leaf blight isolates in the rhizospheric zone of **(A)** Sonalika (susceptible variety), and **(B)** HD2733 (moderately resistant variety). Through absolute quantification, DNA copy number/g soil was calculated for all isolates at 15-day intervals for a period of 3 months. The lines outside the box extend to the highest and lowest observations. The lower and upper hinges correspond to the first and third quartiles. A line across the box represents the sample median and the small black circles represent outliers.

The precise amount of pathogenic inoculum present in the soil, as well as its potential to survive and multiply over time, were investigated by real-time absolute quantification. The data projected a significant difference in the amount of inoculum surviving in the rhizosphere of Sonalika and HD2733. The amount of inoculum present in the soil at all the time points was much higher in Sonalika as compared to HD2733 (Figure 7). On variety Sonalika, isolate LB-7 (1.29 × 10^11^) recorded the maximum and LB-25 (3.41 × 10^9^) recorded the minimum average copy number/g of soil, whereas on HD2733, isolate LB-29 (5.39 × 10^10^) recorded the maximum and LB-25 (5.18 × 10^9^) recorded the minimum average copy number/g of soil. qPCR data demonstrated that all the isolates (belonging to *B. sorokiniana*, *B. spicifera*, and *E. rostratum*) continued to multiply and increase in their numbers on both varieties for 2 months after which all of them recorded a sharp decline in their numbers except isolates LB-50, LB-112, LB-132, and LB-143, which continued to multiply even after 2 months (Supplementary Figures S8a,b). It is noteworthy to observe that the *B. oryzae* isolate exhibited a decline commencing at the 1.5-month mark, persisting in a continuous downward trend thereafter (Figure 7; Supplementary Tables S4a,b).

## 4 Discussion

Wheat is one of the world’s ten most important and extensively cultivated crops. Different regions of the world experience losses in wheat output due to various diseases. One of the pathogens that affect all wheat parts, including seeds, roots, shoots, and leaves, is *B. sorokiniana* (teleomorph, *C. sativus*). Spot blotch disease, which is believed to be a complex of *B. sorokiniana* and *P. tritici-repentis*, is a widespread disease on wheat across many continents (Al-Sadi et al., 2002; Neupane et al., 2010; Al-Sadi, 2016; Devi et al., 2018; Gultyaeva et al., 2018; Gupta et al., 2018; Aggarwal et al., 2022), with losses reaching up to 16–43 percent (Ayana et al., 2018; Devi et al., 2018), particularly in the warm and humid regions of the world. The importance of this disease was recognized in India only during the Green Revolution when most of the introduced semi-dwarf wheat varieties were found to be susceptible to it (Gupta et al., 2018).

The predominant diseases caused by *B. sorokiniana* in wheat are black point, root rot, crown rot, and spot blotch (Kumar et al., 2002; Duveiller et al., 2005; Aggarwal et al., 2009; Burlakoti et al., 2014). Although there have been indications of other pathogens becoming involved, *B. sorokiniana* has remained the center of interest (Bandyopadhyay et al., 2016). In the course of our research, we have come across other pathogenic species that are also contributing significantly to the above-mentioned diseases, which are predominantly attributed to *B. sorokiniana*. We have established by morphological, cultural, molecular, and pathogenicity tests that each isolate employed in the study is a member of the wheat leaf blight/spot blotch complex.

One of the pathogenic species identified in the present study is *E. rostratum*, reported to be associated with black point disease in many countries across the world (Sisterna and Sarandón, 1996; Xu et al., 2018; Li et al., 2019; Somani et al., 2019; Li et al., 2020). It impairs the seed quality and germination in wheat and is regarded as a key source of inoculum for diseases like common root rot and spot blotch. In the present study, *E. rostratum* was isolated from black point infected seeds as well as spot blotch infected leaf samples (Table 1). *E. rostratum* has not been reported as a pathogen of wheat until recently when Korra et al. (2022) proved its pathogenicity on wheat and many other cereals and studied its infection behavior. Through our study, we confirm the association of *E. rostratum* with wheat as a member of the leaf blight/spot blotch complex and highlight its potential as an economically important pathogen. Also, it is noteworthy to mention that the involvement of *E. rostratum* with black point has not been reported earlier in India (Partap et al., 2015; Singh P. et al., 2018; Singh S. et al., 2018). To the best of our knowledge, our study presents the first report of the association of *E. rostratum* with black point disease in wheat in India. The discovery of *E. rostratum* as a novel wheat pathogen has profound implications for agriculture and plant pathology. Its versatile nature, infecting plants, animals, and humans, amplifies the complexity of its impact. With wheat being a predominant staple food crop in India, this finding not only jeopardizes agriculture and related fields but also raises apprehensions regarding human and public health.

**Figure 8:**
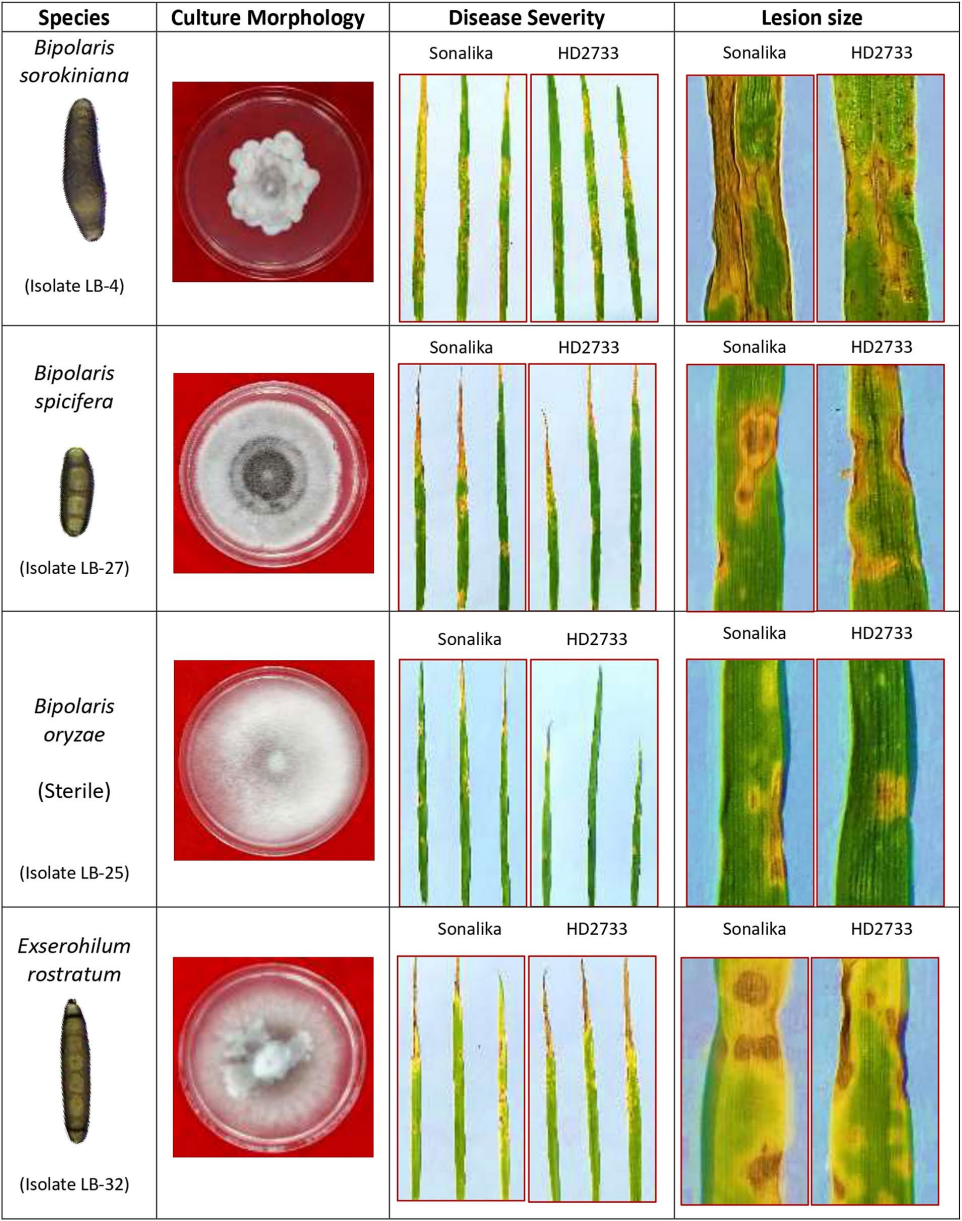
Pictorial illustration of four pathogenic species identified during the study associated with leaf blight/spot blotch complex under Indian climatic conditions.

Morphological characterization has played an important role in characterizing and identifying the isolates in this study. Based on the mycelial growth rate and development pattern, isolates were initially categorized into 2 categories: group I consisted of *B. sorokiniana* isolates with relatively slow growth rate, seldom covering the entire plate (Aggarwal et al., 2009) and group II consisted of *B. spicifera*, *B. oryzae*, and *E. rostratum* isolates with faster growth rates (Biswas and Das, 2018; Dhara et al., 2020; Devi et al., 2021) (Figure 2). Later, based on spore morphology and sporulation studies, group I isolates were identified as *B. sorokiniana* isolates with olive green to brown oblong conidia with prominent scar at the base, having dimensions in the range of 60–120 μm × 15–20 μm and 3–9 septa (Aggarwal et al., 2009; Acharya et al., 2011; Devi et al., 2021). Group II isolates were further subdivided into 2 groups: i) *B. spicifera* isolates which were substantially smaller, had less septation, and were considerably different from *B. sorokiniana* spores (Kumar et al., 2002; Kachkouch et al., 2011; Qostal et al., 2019); and ii) *E. rostratum* isolates with long, thin spores with thick, dark-colored septa at both ends and were very different from the other 2 groups (Ahmadpour et al., 2013; Bunkoed et al., 2014; Sharma et al., 2014; Hernández-Restrepo et al., 2018; Dhara et al., 2020). Further, molecular identification with ITS and GAPDH region sequencing revealed the presence of another pathogenic group, i.e.*, B. oryzae*, represented by a single non-sporulating isolate (LB-25) (Figure 8).

The results of the virulence and pathogenicity studies revealed that all the isolates employed in this investigation were pathogenic on both the wheat varieties, Sonalika and HD2733 (Figure 5). The results clearly indicate that there is substantial variability among isolates and that lesser investigated pathogens are also able to produce comparable disease severity as *B. sorokiniana* (Figure 6). The varied pathogenicity in isolates reveals a spectrum of virulence factors impacting wheat diseases. Notably, some isolates demonstrated pathogenicity on moderately resistant varieties, offering insights into the shift from resistant to moderately resistant status shortly after varietal release (Roy et al., 2023). This understanding is crucial for disease management, emphasizing the necessity of customized approaches such as resistant cultivars and specific fungicides.

The pathogenicity results confirmed that all isolates were pathogenic on wheat and produced symptoms that were indistinguishable from one another. Also, re-isolation from the infected leaves proved the identity of specific isolates. This verifies that the four groups of pathogens isolated in the study, i.e., *B. sorokiniana*, *B. spicifera*, *B. oryzae*, and *E. rostratum*—are all associated with the spot-blotch or leaf blight complex in India (Figure 8). Through this work, we put forth the hypothesis that spot blotch disease exists as a complex, and each group produces comparable disease severity as *B. sorokiniana,* proving them to be economically important and potential threats to various wheat-growing zones in India (Figure 6). The identified pathogens, including *B. spicifera, B. oryzae*, and *E. rostratum*, hold global significance as major plant pathogens affecting Poaceae family crops such as rice, wheat, sugarcane, and maize (Lin et al., 2012; Qostal et al., 2019; Kaboré et al., 2023). *B. spicifera* induces wheat root rot and leaf spot, impacting other graminaceous crops. *B. oryzae*, a serious rice pathogen, exhibits cross-infectivity on wheat and rice (Singh et al., 2021), while *E. rostratum*, versatile across hosts, may pose risks to both plants and potentially animals and humans (Woolhouse and Gaunt, 2007). Understanding these pathogens is vital for global agriculture and public health. The fact that other pathogens, such as *Curvularia lunata*, *Alternaria* spp., and others, were discovered during isolation from infected leaves is also noteworthy. These pathogens were not taken for study as the study was focused on the Helminthosporium group of pathogens.

Based on the results of PCR amplifications with a diagnostic SCAR marker, it seems as though the marker is no longer species-specific in identifying *B. sorokiniana*. However, the marker was still showing negative results for other pathogens which implies that during the course of evolution for over a decade, the closely related isolate population may have undergone some form of genetic exchange event or the pathogenic species have evolved new variants. Such events of genetic exchange and gene flow in asexually reproducing fungi have been reported by several researchers since time long past. Zeigler et al. (1997) demonstrated that *M. grisea* isolates exchanged genetic material in culture and the exchange was evident in field populations. Another example of genetic exchange can be seen in our own pathogen population, when McDonald et al. (2019) reported the transposon mediated *ToxA* gene transfer between *Parastagnospora nodorum*, *P. tritici-repentis,* and *B. sorokiniana*. Aggarwal et al. (2021) also confirmed the presence of *ToxA* gene in Indian *B. sorokiniana* isolate BS112 sharing 100 percent homology with the *ToxA* gene of *P. tritici repentis*. Further studies by Pekarek et al. (2006) in *Aspergillus niger* isolates, Noguchi et al. (2006) in *M. oryzae* isolates, Rosada et al. (2010) in *Colletotrichum lindemuthianum* isolates, Zhao et al. (2021) in *Alternaria solani* isolates using SSR markers, Gourlie et al. (2022) in *P. tritici-repentis* and various others, substantiate the argument and provide opportunities for future research.

The pathogen *B. sorokiniana* affects the majority of wheat parts, including the roots, crown region, stems, leaves, and kernels, in addition to causing considerable production losses. It is reported that the incidence of common root rot is dependent on the soil inoculum present at the time of planting (Boer et al., 1991). Moreover, spot blotch disease, being soil-borne, can be initiated from the inoculum surviving in soil or on straw (Chand et al., 2002; Pandey et al., 2005; Aggarwal et al., 2022). This suggests that management techniques should not only focus on decreasing the presence of the fungus in plant aerial parts but also on the inoculum present in soil (Al-Sadi, 2021). Keeping this in mind, the quantification of soil inoculum using cultural methods and qPCR was carried out in order to explore the persistence and growth of the soil inoculum over time in the presence of two different wheat varieties. Through a serial dilution experiment, it was observed that the pathogenic load was much higher in the susceptible variety, Sonalika, than the moderately resistant variety, HD2733, but the data lacked any development or multiplication pattern, hence, a meaningful conclusion could not be drawn from the quantification data obtained by this experiment. To further track the population of isolates in soil over time during the cropping season, real-time quantification of isolates in soil was performed. Since the SCARBS_600_ species-specific marker, previously used in the study to detect *B. sorokiniana* isolates, was able to detect all leaf blight/spot blotch isolates, we utilized the same markers for studying population dynamics of all 32 isolates in the rhizosphere of Sonalika and HD2733. The findings demonstrated that the soil inoculum multiplies and survives for 2 months thereafter showed a sharp decline. These results are in agreement with the earlier reports of Pratt (2006) and Malaker et al. (2008). qPCR data also substantiates the observation made through serial-dilution experiment that the inoculum surviving in the rhizosphere of the resistant variety was significantly lower than the susceptible variety, Sonalika, for all the isolates at all times during the study (Figure 7), which clearly indicates the impact of using a moderately resistant or resistant variety on inoculum multiplication and survival of pathogenic species associated with the leaf blight complex in wheat. This might be due to the volatile profiles and exudates emitted by the roots of susceptible varieties which may stimulate more fungal growth.

**Figure 9:**
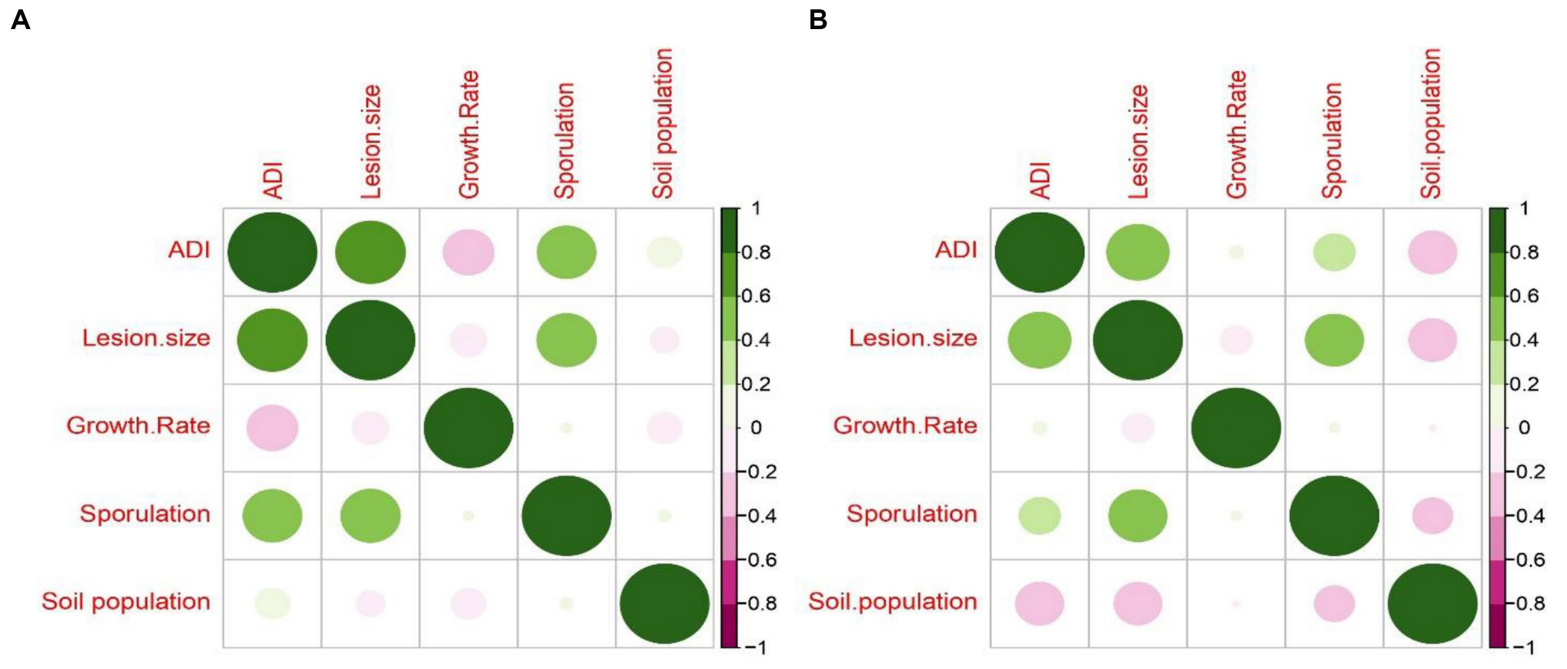
Correlogram representing Pearson’s correlation coefficient for disease severity caused by 32 leaf blight/spot blotch isolates, measured as Average Disease Index (ADI) with fungal growth parameters (lesion size, growth rate on PDA medium, sporulation on PDA medium and soil population) on **(A)** Sonalika (susceptible variety) and **(B)** HD2733 (moderately resistant variety).

It is interesting to note that the average disease severity of all leaf blight/spot blotch isolates showed a strong positive correlation with lesion size/area and relatively no correlation with other growth parameters taken for the study such as growth rate in PDA, sporulation in PDA, and soil population for both varieties (Figure 9). Similar results have been found in the case of *Fusarium udum* isolates where mycelial growth and sporulation were found to have no correlation with isolate virulence (Kiprop et al., 2002). The lack of correlation between disease severity and growth rate on PDA corresponds with the fact that the isolates with a lesser growth rate (Group 1: *B. sorokiniana*) produced the maximum disease severity on both varieties. Furthermore, the sterile isolate LB-24 with zero sporulation caused more disease severity on both varieties than the highly sporulating isolate LB-26. Bashyal et al. (2010) observed a negative correlation between melanin content and aggressiveness in *B. sorokiniana* isolates. White (sterile) isolates were more aggressive on barley than black sporulating ones. It was proposed that constitutive melanization may reduce aggressiveness but enhance survival fitness, potentially explaining the prevalence of black sporulating isolates in nature. The fact that the soil population of leaf blight isolates did not share any positive or negative correlation with any of the parameters may indicate the presence of different mechanisms operating for different lifestyles of the pathogens (Figure 9).

## 5 Conclusion

This study reexamined the pathogenic flora associated with the wheat leaf blight/spot blotch disease complex in India, highlighting the roles of *B. spicifera*, *E. rostratum*, *B. oryzae*, and *B. sorokiniana* in the disease complex, which was largely represented solely by *B. sorokiniana*. Furthermore, *E. rostratum* is closely linked to the spot blotch and black point disease complex, as it was found in both infected seeds and leaves. *B. oryzae*’s involvement in spot blotch disease is established, supporting cross-infectivity findings previously reported from our lab. This research underscores the impact of these infections on wheat quality and output in Indian conditions. Looking ahead, further exploration of control strategies and resistant cultivars is crucial for mitigating their effects and ensuring sustainable wheat production.

## Data availability statement

The datasets presented in this study can be found in online repositories. The names of the repository/repositories and accession number(s) can be found in the article/Supplementary material.

## Author contributions

SaA: Conceptualization, Data curation, Formal analysis, Investigation, Methodology, Software, Validation, Visualization, Writing – original draft, Writing – review & editing. RA: Conceptualization, Data curation, Funding acquisition, Project administration, Resources, Supervision, Validation, Visualization, Writing – review & editing. BB: Conceptualization, Project administration, Supervision, Validation, Writing – review & editing. MG: Supervision, Validation, Writing – review & editing. MS: Project administration, Supervision, Validation, Writing – review & editing. ShA: Investigation, Methodology, Writing – review & editing.

## Footnotes

^1^ https://www.fao.org/faostat/en/#home

^2^ https://www.cimmyt.org/annual-reports/cimmyt-annual-report-2013/

^3^ https://iiwbr.icar.gov.in/

^4^ http://www.ncbi.nlm.nih.gov/

^5^ https://www.megasoftware.net/

^6^ https://cran.r-project.org/bin/windows/base/
