# Corrigendum: Unraveling the dynamics of wheat leaf blight complex: isolation, characterization, and insights into pathogen population under Indian conditions

**DOI:** 10.3389/fmicb.2024.1409209

**Published:** 2024-04-12

**Authors:** Sanghmitra Aditya, Rashmi Aggarwal, Bishnu Maya Bashyal, Malkhan Singh Gurjar, Mahender Singh Saharan, Shweta Aggarwal

**Affiliations:** Fungal Molecular Biology Laboratory, Division of Plant Pathology, ICAR-Indian Agricultural Research Institute, New Delhi, India

**Keywords:** leaf blight/spot blotch complex, wheat, soil population dynamics, absolute quantification, *Bipolaris spicifera*, *Exserohilum rostratum*

In the published article, there was an error in the **Funding** statement. The assistance from “ICAR-Emeritus Scientist Project” (project code: 16-100), which provided financial assistance for gene sequencing to Sanghmitra Aditya and Rashmi Aggarwal, was erroneously omitted. The correct **Funding** statement appears below:

“The author(s) declare that financial support was received for the research, authorship, and/or publication of this article. SaA and RA gratefully acknowledge the funding provided for gene sequencing by the “Emeritus Scientist Project” sanction letter no. 9(2)/2023-ES-HRD (Project Code: 16-100) of Indian Council of Agricultural Research, New Delhi”.

The authors apologize for this error and state that this does not change the scientific conclusions of the article in any way. The original article has been updated.

