# Supplementary material for: Unraveling the dynamics of wheat leaf blight complex: isolation, characterization, and insights into pathogen population under Indian conditions
Source: Front Microbiol. 2024 Feb 21;15:1287721. doi: 10.3389/fmicb.2024.1287721 (PMC10915091; doi:10.3389/fmicb.2024.1287721)
Supplement: Supplementary file 1 [file Data_Sheet_1.docx]

**Supplementary Figure S1:** Leaf blight/spot blotch isolates collected from six wheat-growing zones in India during the *Rabi* season from 2020-2022.

**
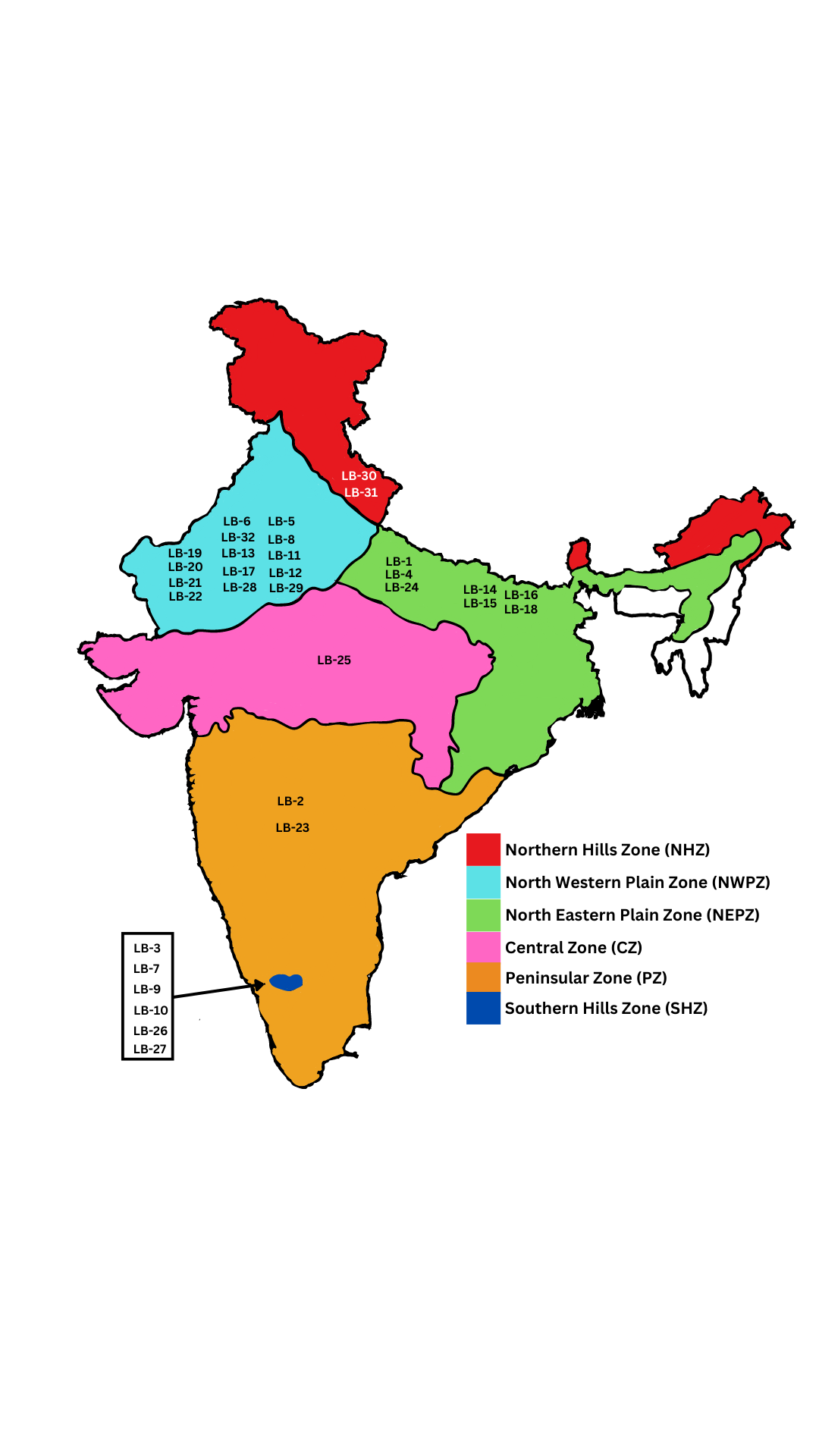
**

**Supplementary Figure S2:** Schematic standard curve of a dilution series, plotting *C_T_* values over log template concentrations

**Supplementary Figure S3**: 7-day-old purified Mono-conidial cultures of leaf blight/spot blotch isolates collected during the study on potato dextrose agar medium


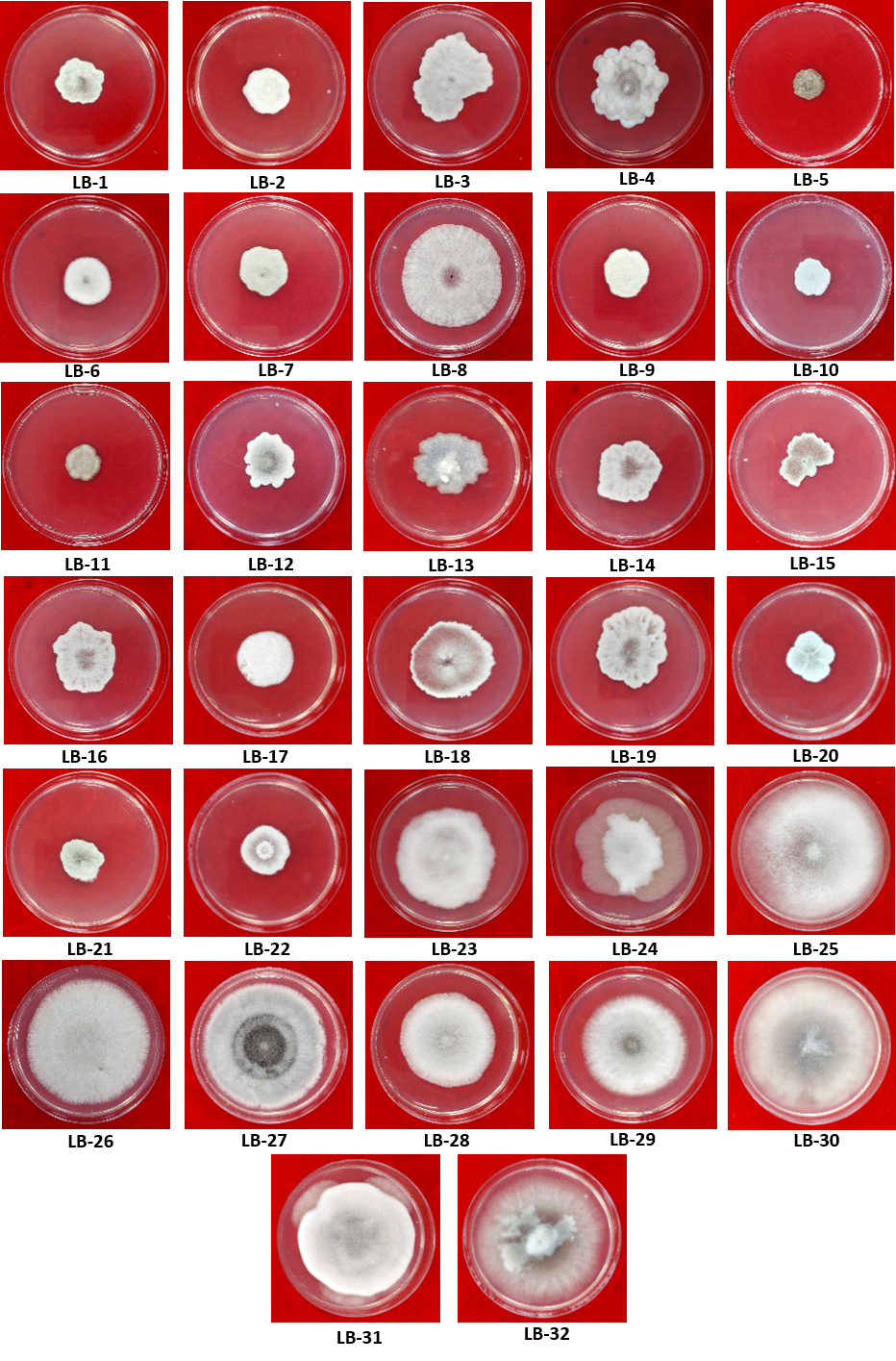


**Supplementary Figure S4**: Spore morphology studies of leaf blight/spot blotch isolates infecting wheat under Indian climatic conditions. Pictures are taken at 40X magnification

| **LB-1** | **LB-2** | **LB-3** | **LB-4** | **LB-5** | **LB-6** |
| --- | --- | --- | --- | --- | --- |
| **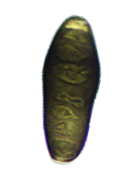** | **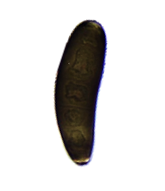** | **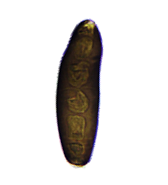** | **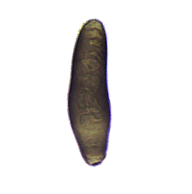** | **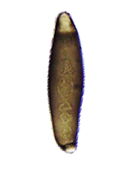** | **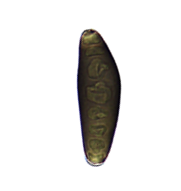** |
| **LB-7** | **LB-8** | **LB-9** | **LB-10** | **LB-11** | **LB-12** |
| **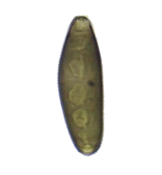** | **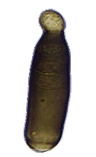** | **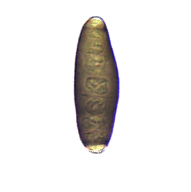** | **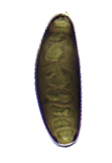** | **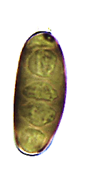** | **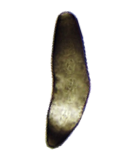** |
| **LB-13** | **LB-14** | **LB-15** | **LB-16** | **LB-17** | **LB-18** |
| **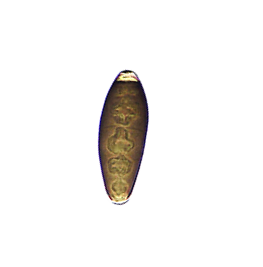** | **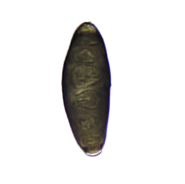** | **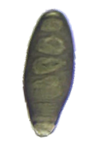** | **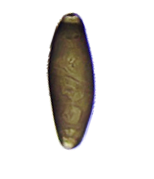** | **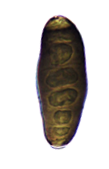** | **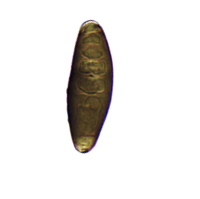** |
| **LB-19** | **LB-20** | **LB-21** | **LB-22** | **LB-26** | **LB-27** |
| **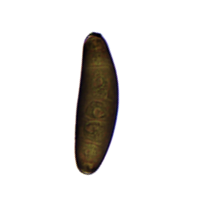** | **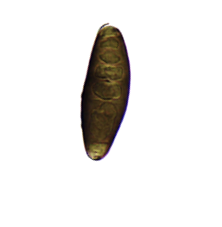** | **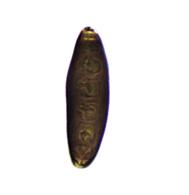** | **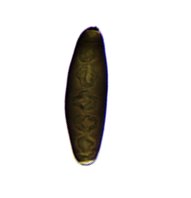** | **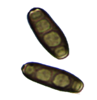** | **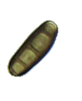** |
| **LB-28** | **LB-29** | **LB-30** | **LB-31** | **LB-32** |  |
| **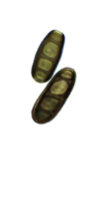** | **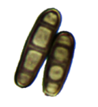** | **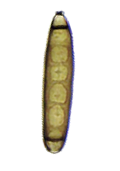** | **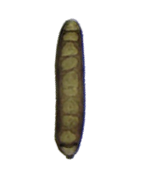** | **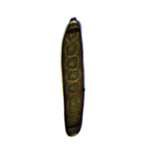** |  |

*Note: Isolates LB-23, LB-24, and LB-25 are sterile cultures and hence, their spore morphology could not be determined.

**Supplementary Figure S5:** PCR amplification pattern for leaf blight/spot blotch isolates using SCRABS_600_ marker F/R. Only the 600 bp specific fragment is amplified. M, 1 kb DNA ladder. PCR reactions were done in triplicate using DNA extracted from fungal mycelial mats.


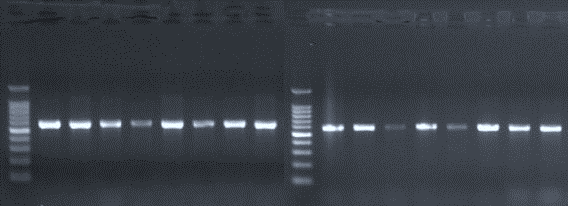


600 bp

LB-1

LB-2

LB-3

LB-4

LB-5

LB-6

LB-7

LB-8

LB-9

LB-10

LB-11

LB-12

LB-13

LB-14

LB-15

LB-16

M

M


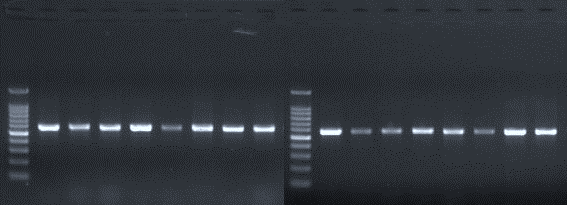


600 bp

LB-17

LB-18

LB-19

LB-20

LB-21

LB-22

LB-23

LB-24

LB-25

LB-26

LB-27

LB-28

LB-29

LB-30

LB-31

LB-32

M

M

**Supplementary Figure S6**: Concatenated phylogenetic tree constructed from maximum likelihood method based on combined ITS and GAPDH sequences of 32 isolates inferred with RAxML based on GTR + Gamma model.


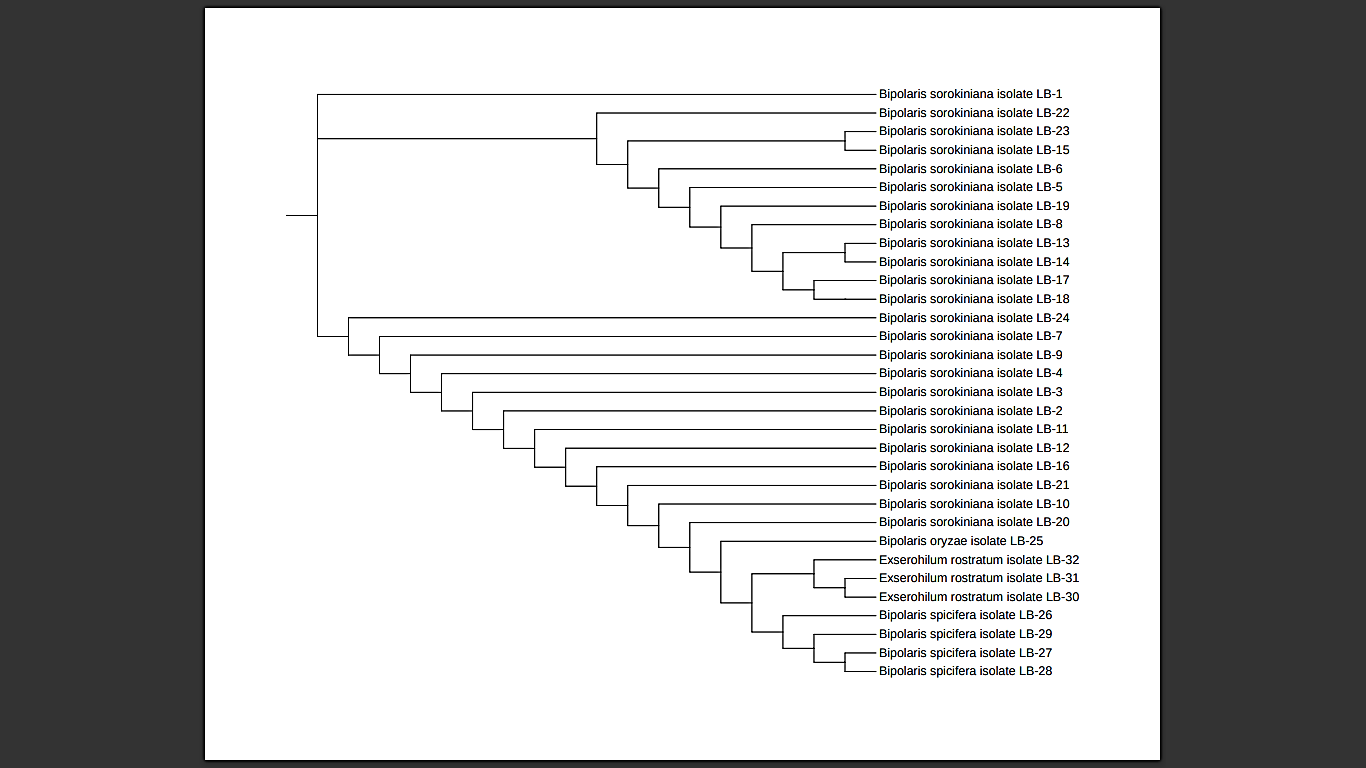


**Supplementary Figure S7**: Pathogenicity studies conducted with 32 leaf blight/spot blotch isolates on two wheat varieties, Sonalika (susceptible) and HD2733 (moderately resistant), from 2020-2022 under polyhouse conditions

|  | **Sonalika** | **HD2733** |  | **Sonalika** | **HD2733** |  | **Sonalika** | **HD2733** |
| --- | --- | --- | --- | --- | --- | --- | --- | --- |
| LB-1 | 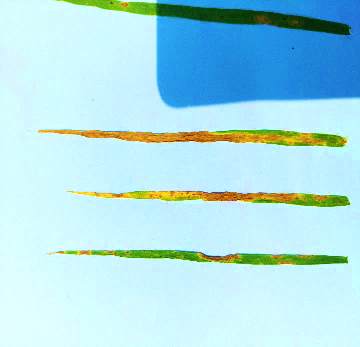 | 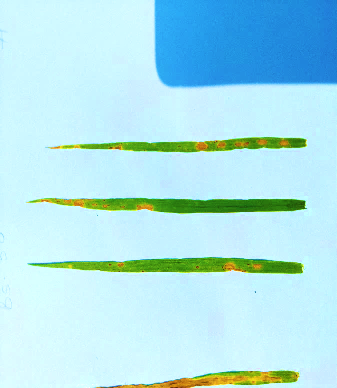 | LB-7 | 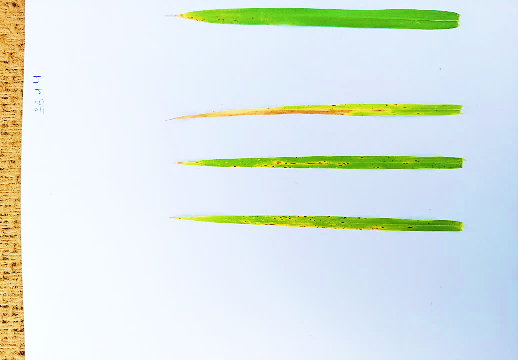 | 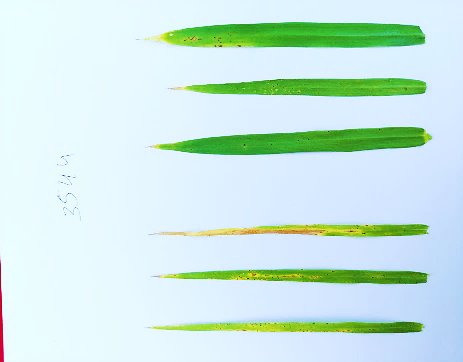 | LB-13 | 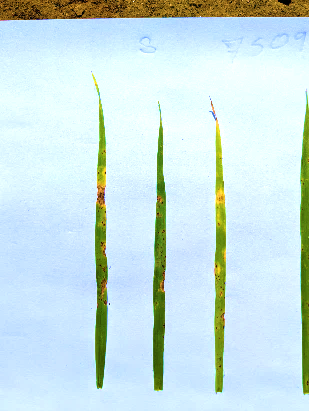 | 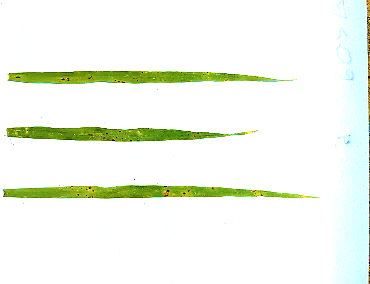 |
| LB-2 | 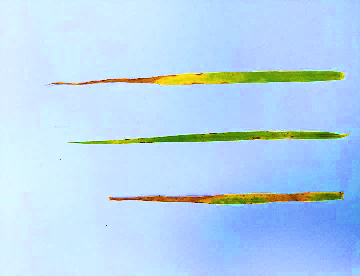 | 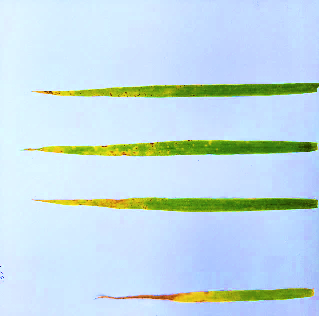 | LB-8 | 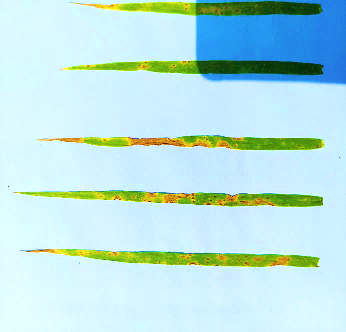 | 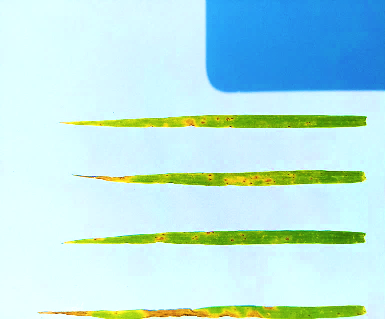 | LB-14 | 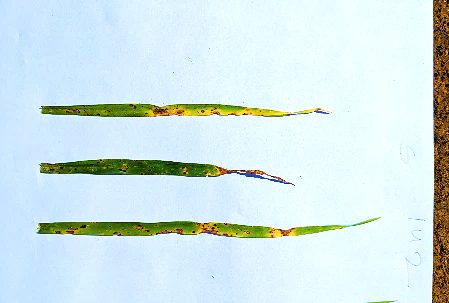 | 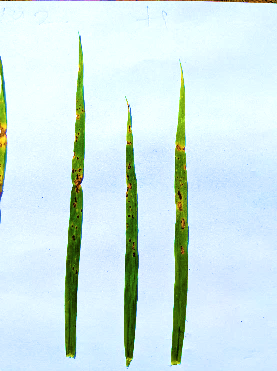 |
| LB-3 | 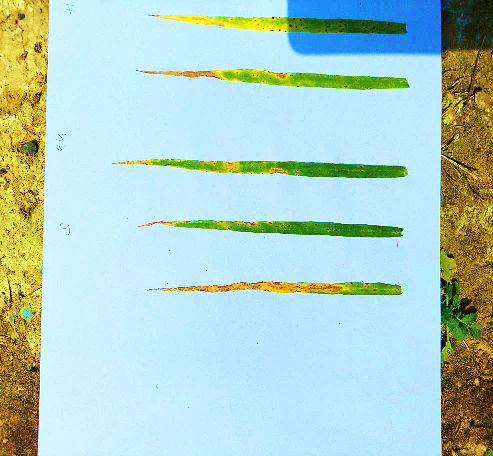 | 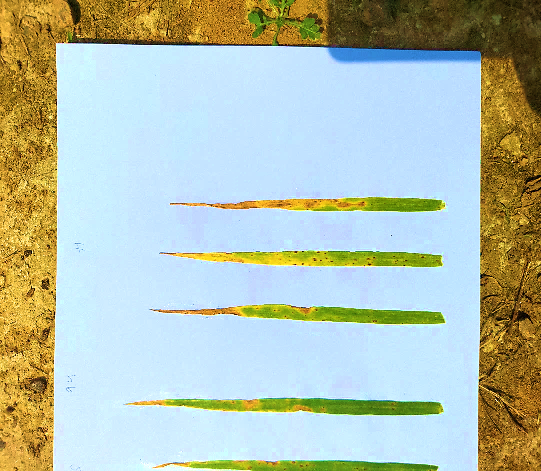 | LB-9 | 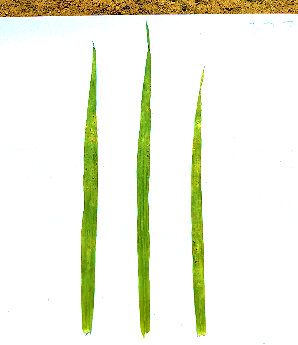 | 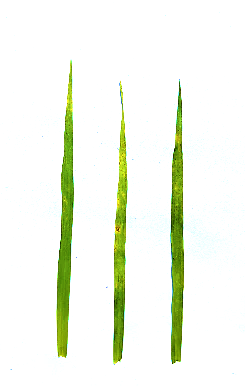 | LB-15 | 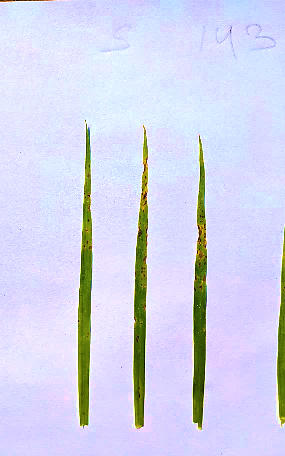 | 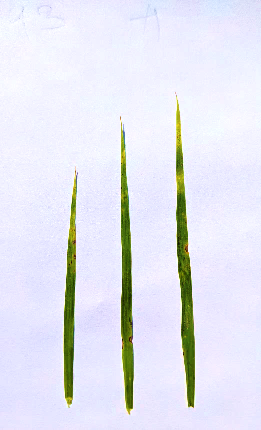 |
| LB-4 | 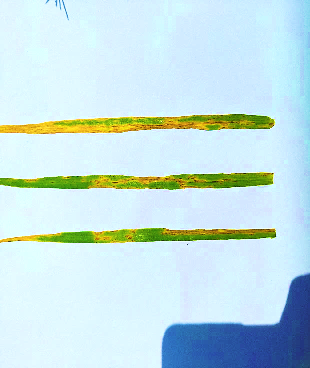 | 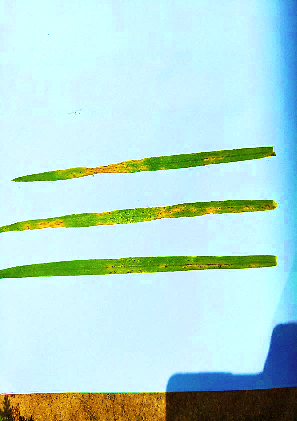 | LB-10 | 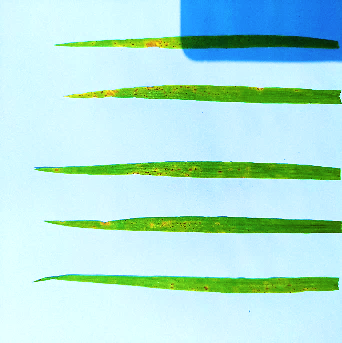 | 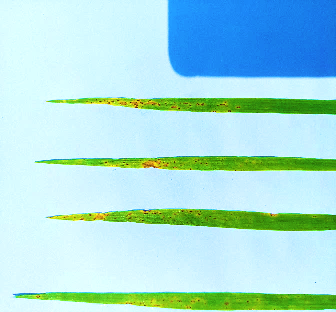 | LB-16 | 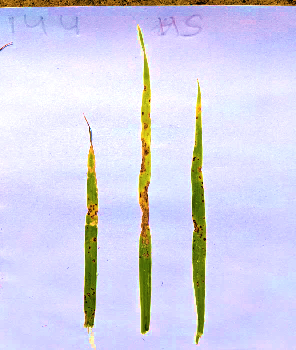 | 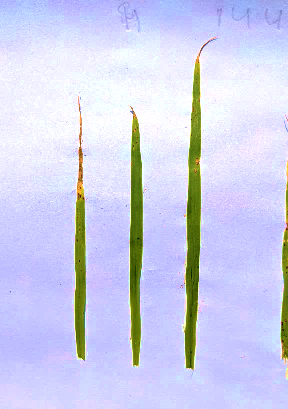 |
| LB-5 | 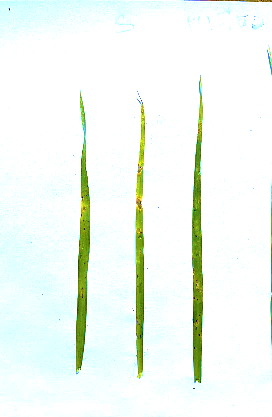 | 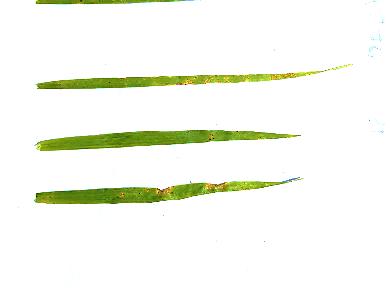 | LB-11 | 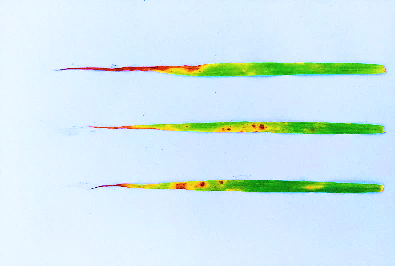 | 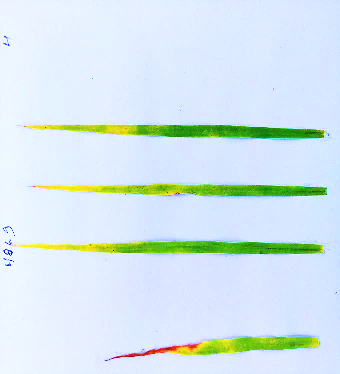 | LB-17 | 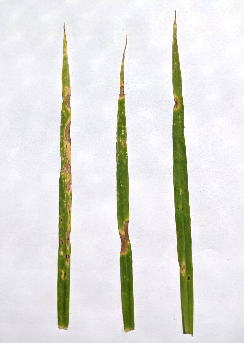 | 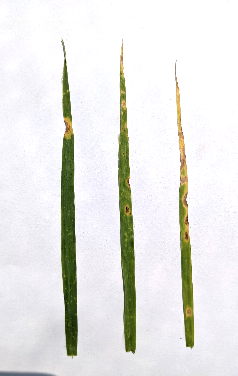 |
| LB-6 | 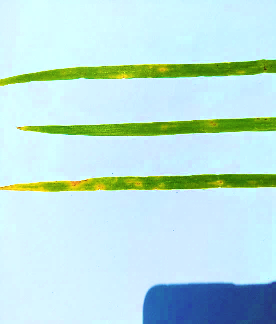 | 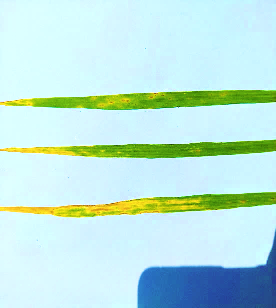 | LB-12 | 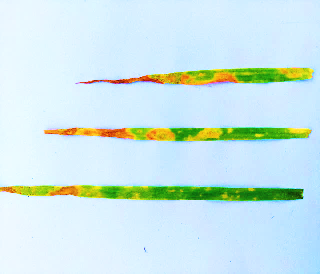 | 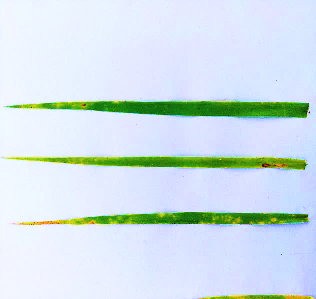 | LB-18 | 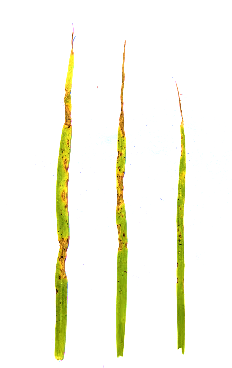 | 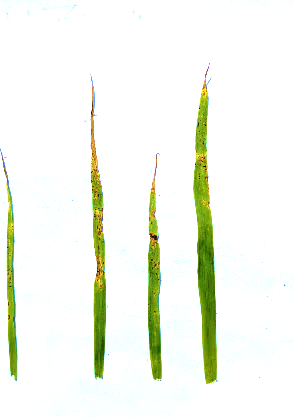 |

| LB-19 | 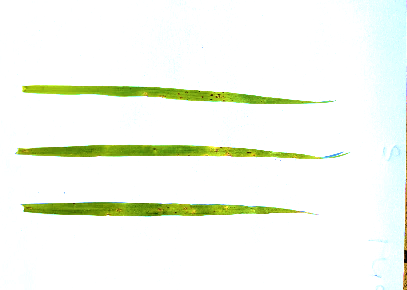 | 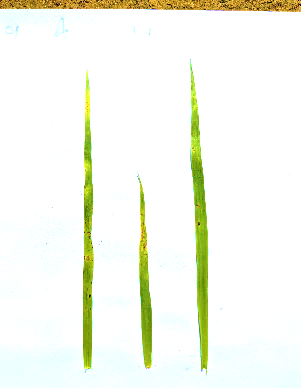 | LB-24 | 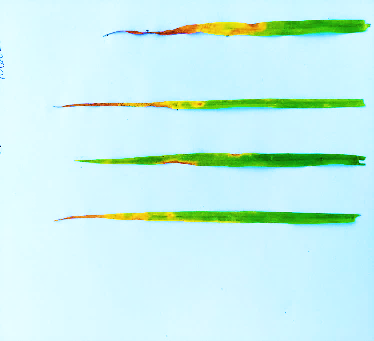 | 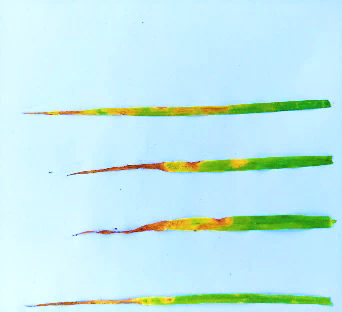 | LB-29 | 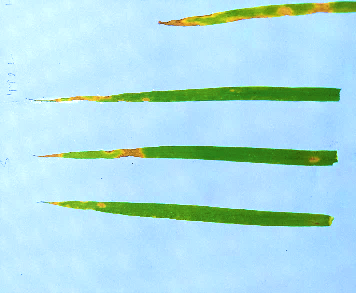 | 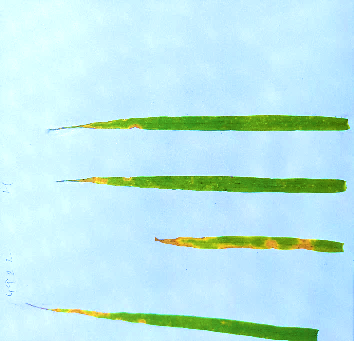 |
| --- | --- | --- | --- | --- | --- | --- | --- | --- |
| LB-20 | 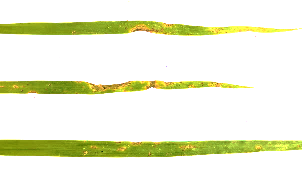 | 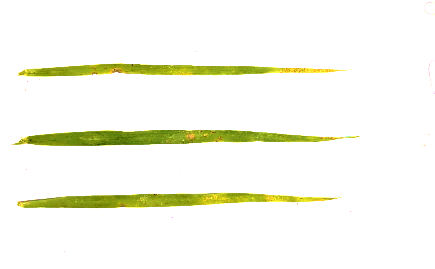 | LB-25 | 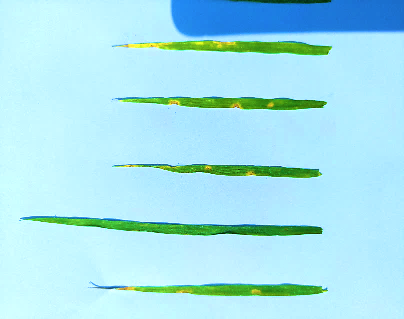 | 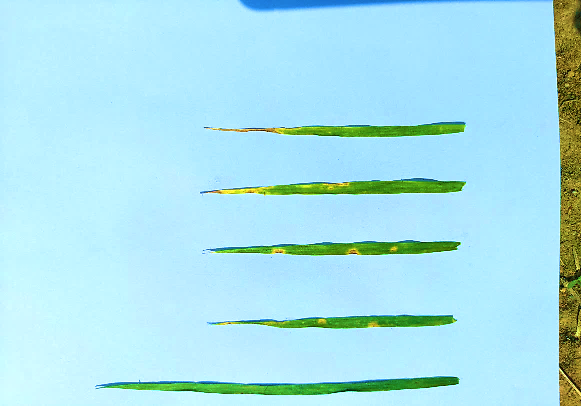 | LB-30 | 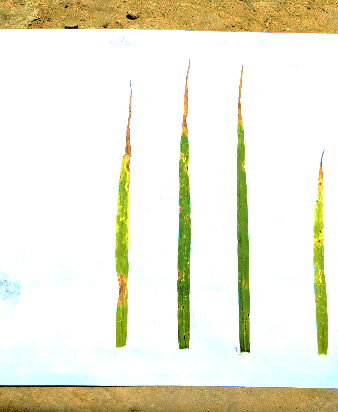 | 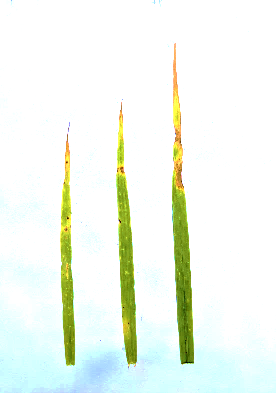 |
| LB-21 | 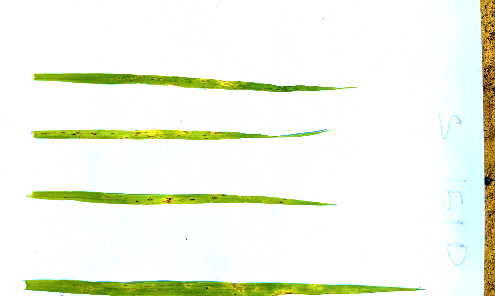 | 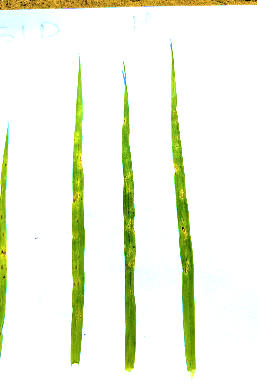 | LB-26 | 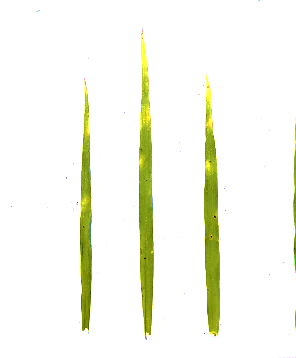 | 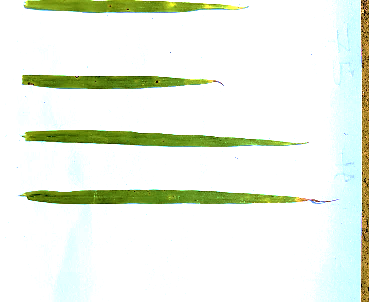 | LB-31 | 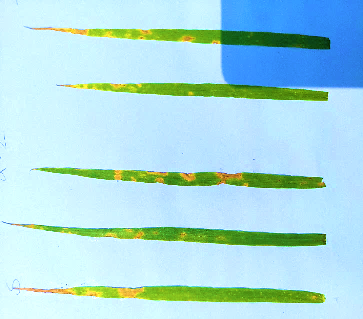 | 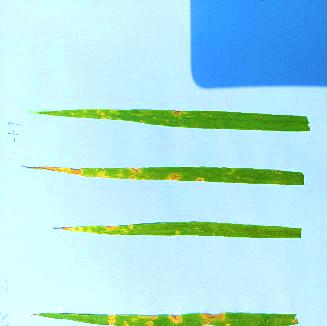 |
| LB-22 | 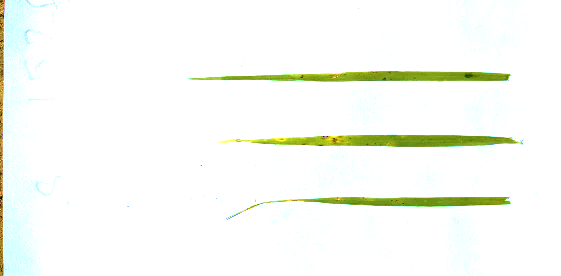 | 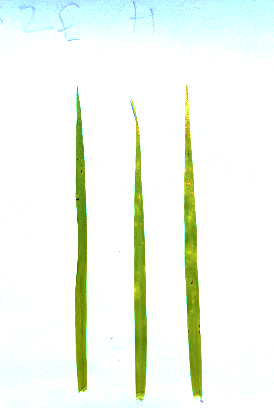 | LB-27 | 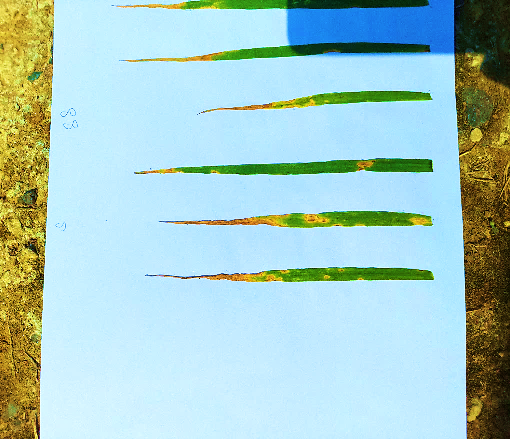 | 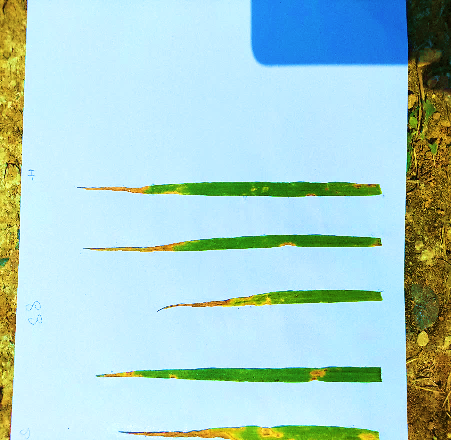 | LB-32 | 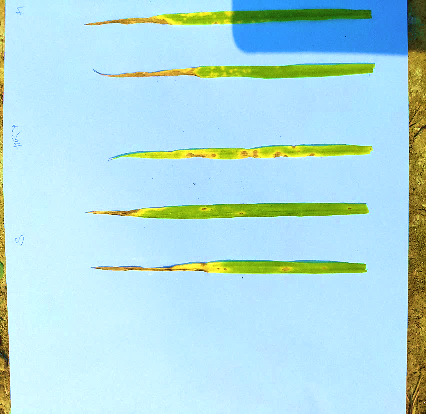 | 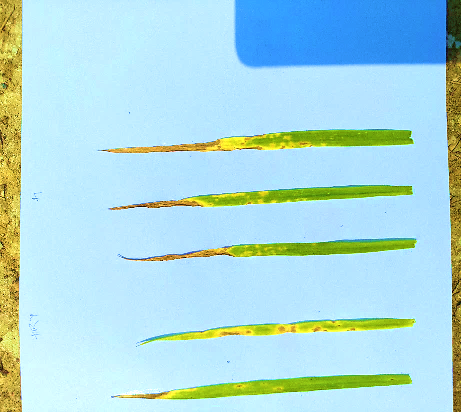 |
| LB-23 | 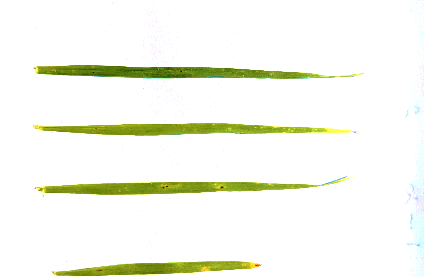 | 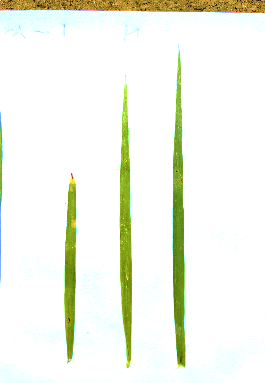 | LB-28 | 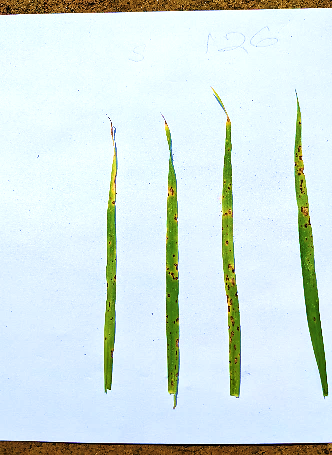 | 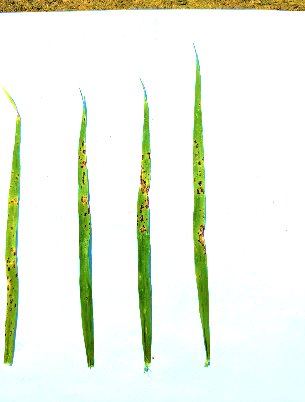 |  |  |  |

**Supplementary Figure S8a:** Soil population dynamics of 32 leaf blight/spot blotch isolates in the rhizospheric zone of wheat variety Sonalika

**Supplementary Figure S8b:** Soil population dynamics of 32 leaf blight/spot blotch isolates in the rhizospheric zone of wheat variety HD2733

**Supplementary Table S1**: Radial mycelial growth rate of leaf blight isolates on potato dextrose agar medium recorded at 3-day intervals until 15 days

| **S.No** | **Isolate** | **Radial mycelial growth (mm)** | | | | | **Average Growth Rate**  **(mm/day)** |
| --- | --- | --- | --- | --- | --- | --- | --- |
|  |  | **3 Days** | **6 days** | **9 days** | **12 days** | **15 days** |  |
| 1 | LB-1 | 14.35 | 17.50 | 21.50 | 23.45 | 26.85 | 1.79 |
| 2 | LB-2 | 10.75 | 15.15 | 17.00 | 19.00 | 24.50 | 1.63 |
| 3 | LB-3 | 15.20 | 41.50 | 45.00 | 46.00 | 49.00 | 3.27 |
| 4 | LB-4 | 23.25 | 46.40 | 50.90 | 56.80 | 59.60 | 3.97 |
| 5 | LB-5 | 11.00 | 14.65 | 17.00 | 19.00 | 20.35 | 1.36 |
| 6 | LB-6 | 4.95 | 11.50 | 17.50 | 24.00 | 25.20 | 1.68 |
| 7 | LB-7 | 13.10 | 18.55 | 19.00 | 19.50 | 21.95 | 1.46 |
| 8 | LB-8 | 25.00 | 56.50 | 61.75 | 65.65 | 78.35 | 5.22 |
| 9 | LB-9 | 12.95 | 17.00 | 23.00 | 26.05 | 27.05 | 1.80 |
| 10 | LB-10 | 7.00 | 8.95 | 12.00 | 13.45 | 14.95 | 1.00 |
| 11 | LB-11 | 12.40 | 21.35 | 35.00 | 48.35 | 50.60 | 3.37 |
| 12 | LB-12 | 4.60 | 5.00 | 12.50 | 17.00 | 18.15 | 1.21 |
| 13 | LB-13 | 5.00 | 11.00 | 12.35 | 13.00 | 18.55 | 1.24 |
| 14 | LB-14 | 13.35 | 17.50 | 23.00 | 27.35 | 30.30 | 2.02 |
| 15 | LB-15 | 19.50 | 30.25 | 33.00 | 36.90 | 38.75 | 2.58 |
| 16 | LB-16 | 13.00 | 20.05 | 25.05 | 26.95 | 28.80 | 1.92 |
| 17 | LB-17 | 13.00 | 23.00 | 32.30 | 41.10 | 42.35 | 2.82 |
| 18 | LB-18 | 9.00 | 27.60 | 31.00 | 36.00 | 37.25 | 2.48 |
| 19 | LB-19 | 16.00 | 21.50 | 25.65 | 28.30 | 29.00 | 1.93 |
| 20 | LB-20 | 12.50 | 19.65 | 23.25 | 25.00 | 26.00 | 1.73 |
| 21 | LB-21 | 9.00 | 13.05 | 15.45 | 16.80 | 18.95 | 1.26 |
| 22 | LB-22 | 10.00 | 15.50 | 17.35 | 22.30 | 25.15 | 1.68 |
| 23 | LB-23 | 14.30 | 37.00 | 54.85 | 79.80 | 87.00 | 5.80 |
| 24 | LB-24 | 25.05 | 58.95 | 73.45 | 85.65 | 88.00 | 5.87 |
| 25 | LB-25 | 33.95 | 65.10 | 77.50 | 85.10 | 88.00 | 5.87 |
| 26 | LB-26 | 27.45 | 54.60 | 79.55 | 85.00 | 88.00 | 5.87 |
| 27 | LB-27 | 28.05 | 57.00 | 77.50 | 79.80 | 88.00 | 5.87 |
| 28 | LB-28 | 23.20 | 56.10 | 74.50 | 83.00 | 88.00 | 5.87 |
| 29 | LB-29 | 29.50 | 54.40 | 71.50 | 83.95 | 88.00 | 5.87 |
| 30 | LB-30 | 25.00 | 42.50 | 83.50 | 88.00 | 88.00 | 5.87 |
| 31 | LB-31 | 26.50 | 59.00 | 74.50 | 81.00 | 88.00 | 5.87 |
| 32 | LB-32 | 25.15 | 39.50 | 83.00 | 84.85 | 88.00 | 5.87 |
|  | C.D. | 0.86 | 1.35 | 1.92 | 1.39 | 1.33 | 3.6 |
|  | SE(m) | 0.30 | 0.47 | 0.41 | 0.48 | 0.46 | 1.28 |
|  | SE(d) | 0.42 | 0.66 | 0.58 | 0.68 | 0.65 | 1.81 |
|  | C.V. | 2.52 | 2.11 | 1.42 | 1.46 | 1.31 |  |

**Supplementary Table S2:** List of 32 leaf blight/spot blotch isolates collected from six wheat growing zones in India. The isolate name, their identified species, and GenBank Accessions of the internal transcribed spacer (ITS) sequences and Glyceraldehyde-3-phosphate dehydrogenase (GAPDH) protein sequences, expressed at >99% similarity index, are provided.

| **S.No** | **Isolate name** | **Species** | **ITS Accession no.** | **GAPDH Accession no.** |
| --- | --- | --- | --- | --- |
| 1 | LB-1 | *Bipolaris sorokiniana* | OQ845799 | OR260680 |
| 2 | LB-2 | *Bipolaris sorokiniana* | OQ845800 | OR260681 |
| 3 | LB-3 | *Bipolaris sorokiniana* | OQ845801 | OR260682 |
| 4 | LB-4 | *Bipolaris sorokiniana* | OQ845802 | OR260683 |
| 5 | LB-5 | *Bipolaris sorokiniana* | OQ845803 | OR260684 |
| 6 | LB-6 | *Bipolaris sorokiniana* | OQ845804 | OR260685 |
| 7 | LB-7 | *Bipolaris sorokiniana* | OQ845805 | OR260686 |
| 8 | LB-8 | *Bipolaris sorokiniana* | OQ845806 | OR260687 |
| 9 | LB-9 | *Bipolaris sorokiniana* | OQ845807 | OR260688 |
| 10 | LB-10 | *Bipolaris sorokiniana* | OQ845808 | OR260689 |
| 11 | LB-11 | *Bipolaris sorokiniana* | OQ845809 | OR260690 |
| 12 | LB-12 | *Bipolaris sorokiniana* | OQ845810 | OR260691 |
| 13 | LB-13 | *Bipolaris sorokiniana* | OQ845811 | OR260692 |
| 14 | LB-14 | *Bipolaris sorokiniana* | OQ845812 | OR260693 |
| 15 | LB-15 | *Bipolaris sorokiniana* | OQ845813 | OR260694 |
| 16 | LB-16 | *Bipolaris sorokiniana* | OQ845814 | OR260695 |
| 17 | LB-17 | *Bipolaris sorokiniana* | OQ845815 | OR260696 |
| 18 | LB-18 | *Bipolaris sorokiniana* | OQ845816 | OR260697 |
| 19 | LB-19 | *Bipolaris sorokiniana* | OQ845817 | OR260698 |
| 20 | LB-20 | *Bipolaris sorokiniana* | OQ845818 | OR260699 |
| 21 | LB-21 | *Bipolaris sorokiniana* | OQ845819 | OR260700 |
| 22 | LB-22 | *Bipolaris sorokiniana* | OQ845820 | OR260701 |
| 23 | LB-23 | *Bipolaris sorokiniana* | OQ845821 | OR260702 |
| 24 | LB-24 | *Bipolaris sorokiniana* | OQ845822 | OR260703 |
| 25 | LB-25 | *Bipolaris oryzae* | OQ845823 | OR260675 |
| 26 | LB-26 | *Bipolaris spicifera* | OQ845824 | OR260676 |
| 27 | LB-27 | *Bipolaris spicifera* | OQ845825 | OR260677 |
| 28 | LB-28 | *Bipolaris spicifera* | OQ845826 | OR260678 |
| 29 | LB-29 | *Bipolaris spicifera* | OQ845827 | OR260679 |
| 30 | LB-30 | *Exserohilum rostratum* | OQ845828 | OR260672 |
| 31 | LB-31 | *Exserohilum rostratum* | OQ845829 | OR260673 |
| 32 | LB-32 | *Exserohilum rostratum* | OQ845830 | OR260674 |

**Supplementary Table S3a:** Quantification of leaf blight/spot blotch isolates through serial dilution method in the rhizosphere of susceptible wheat variety Sonalika at 15-day intervals for a period of 3 months. (*Unit: 1 × 10^3^ colonies/g of soil)

| **S.No** | **Isolate** | **I** | **II** | **III** | **IV** | **V** |
| --- | --- | --- | --- | --- | --- | --- |
| 1 | LB-1 | 10.2 | 20.0 | 16.5 | 32.0 | 42.5 |
| 2 | LB-2 | 12.2 | 15.0 | 15.5 | 3.5 | 17.0 |
| 3 | LB-3 | 10.0 | 5.0 | 7.0 | 0.0 | 0.0 |
| 4 | LB-4 | 4.0 | 2.0 | 3.0 | 1.0 | 3.0 |
| 5 | LB-5 | 8.0 | 13.5 | 19.0 | 24.2 | 20.5 |
| 6 | LB-6 | 12.0 | 14.5 | 11.0 | 9.0 | 3.0 |
| 7 | LB-7 | 13.0 | 19.5 | 24.5 | 20.5 | 15.0 |
| 8 | LB-8 | 10.0 | 3.0 | 0.0 | 7.0 | 6.0 |
| 9 | LB-9 | 10.5 | 11.0 | 8.0 | 10.5 | 22.5 |
| 10 | LB-10 | 9.0 | 17.0 | 32.0 | 21.5 | 13.0 |
| 11 | LB-11 | 7.0 | 11.0 | 14.0 | 3.0 | 9.5 |
| 12 | LB-12 | 8.0 | 5.0 | 7.0 | 2.0 | 5.0 |
| 13 | LB-13 | 7.0 | 3.0 | 12.0 | 9.5 | 25.0 |
| 14 | LB-14 | 13.0 | 23.5 | 30.0 | 36.0 | 25.0 |
| 15 | LB-15 | 9.0 | 8.5 | 14.0 | 17.0 | 10.0 |
| 16 | LB-16 | 13.0 | 18.0 | 20.0 | 23.5 | 35.0 |
| 17 | LB-17 | 10.0 | 8.0 | 7.0 | 3.0 | 3.0 |
| 18 | LB-18 | 9.0 | 15.0 | 17.0 | 17.5 | 23.0 |
| 19 | LB-19 | 10.0 | 11.0 | 15.0 | 18.5 | 11.0 |
| 20 | LB-20 | 11.0 | 14.0 | 15.5 | 20.0 | 12.0 |
| 21 | LB-21 | 12.0 | 9.0 | 10.0 | 9.5 | 12.0 |
| 22 | LB-22 | 12.0 | 18.5 | 24.5 | 37.5 | 17.5 |
| 23 | LB-23 | 8.0 | 15.5 | 18.0 | 24.5 | 5.0 |
| 24 | LB-24 | 12.0 | 14.5 | 11.0 | 6.5 | 2.0 |
| 25 | LB-25 | 9.0 | 16.0 | 23.5 | 20.0 | 4.0 |
| 26 | LB-26 | 12.5 | 15.5 | 19.0 | 12.0 | 20.5 |
| 27 | LB-27 | 18.0 | 30.5 | 49.0 | 42.0 | 11.0 |
| 28 | LB-28 | 13.0 | 11.0 | 17.0 | 13.5 | 11.0 |
| 29 | LB-29 | 13.0 | 20.0 | 40.0 | 33.0 | 17.5 |
| 30 | LB-30 | 12.0 | 7.5 | 15.0 | 19.5 | 10.5 |
| 31 | LB-31 | 10.0 | 7.0 | 3.5 | 10.0 | 9.0 |
| 32 | LB-32 | 12.0 | 14.5 | 8.0 | 5.0 | 3.0 |

**Supplementary Table S3b:** Quantification of leaf blight/spot blotch isolates through serial dilution method in the rhizosphere of moderately resistant wheat variety HD2733 at 15-day intervals for a period of 3 months (*Unit: 1 × 10^3^ colonies/g of soil)

| **S.No** | **Isolate** | **I** | **II** | **III** | **IV** | **V** |
| --- | --- | --- | --- | --- | --- | --- |
| 1 | LB-1 | 9.0 | 10.5 | 15.5 | 18.5 | 22.0 |
| 2 | LB-2 | 10.0 | 8.5 | 10.5 | 2.5 | 10.0 |
| 3 | LB-3 | 9.0 | 12.0 | 7.0 | 1.0 | 2.0 |
| 4 | LB-4 | 2.0 | 0.0 | 1.0 | 2.0 | 2.0 |
| 5 | LB-5 | 6.0 | 9.0 | 5.0 | 8.5 | 8.0 |
| 6 | LB-6 | 8.0 | 5.0 | 9.0 | 11.0 | 2.0 |
| 7 | LB-7 | 11.0 | 17.0 | 20.5 | 18.0 | 13.0 |
| 8 | LB-8 | 9.0 | 13.0 | 6.0 | 3.0 | 2.0 |
| 9 | LB-9 | 8.0 | 10.0 | 11.5 | 7.5 | 12.5 |
| 10 | LB-10 | 12.0 | 19.5 | 24.0 | 13.5 | 8.6 |
| 11 | LB-11 | 9.0 | 12.0 | 7.0 | 2.0 | 3.5 |
| 12 | LB-12 | 6.0 | 3.0 | 2.5 | 2.0 | 2.5 |
| 13 | LB-13 | 9.0 | 3.5 | 8.0 | 7.5 | 23 |
| 14 | LB-14 | 11.0 | 12.0 | 13.5 | 20.0 | 8.5 |
| 15 | LB-15 | 8.0 | 13.0 | 20.5 | 4.5 | 6.5 |
| 16 | LB-16 | 11.0 | 14.0 | 20.5 | 16.5 | 15.0 |
| 17 | LB-17 | 8.0 | 6.0 | 2.5 | 2.5 | 1.0 |
| 18 | LB-18 | 10.0 | 13.0 | 13.5 | 15.0 | 25.0 |
| 19 | LB-19 | 11.0 | 14.5 | 16.0 | 8.5 | 8.0 |
| 20 | LB-20 | 8.0 | 11.5 | 14.5 | 10.0 | 14.5 |
| 21 | LB-21 | 13.0 | 14.5 | 18.0 | 17.0 | 11.5 |
| 22 | LB-22 | 11.0 | 14.0 | 18.5 | 12.0 | 18.5 |
| 23 | LB-23 | 9.5 | 11.0 | 11.5 | 8.0 | 3.0 |
| 24 | LB-24 | 11.0 | 9.0 | 12.0 | 6.0 | 8.0 |
| 25 | LB-25 | 11.0 | 14.5 | 9.0 | 12.0 | 2.5 |
| 26 | LB-26 | 13.0 | 11.5 | 14.5 | 11.0 | 6.0 |
| 27 | LB-27 | 13.0 | 11.0 | 11.5 | 10.0 | 8.5 |
| 28 | LB-28 | 8.0 | 9.0 | 11.0 | 7.5 | 3.5 |
| 29 | LB-29 | 15.0 | 17.5 | 8.0 | 13.0 | 14.0 |
| 30 | LB-30 | 5.0 | 4.5 | 9.0 | 11.0 | 13.0 |
| 31 | LB-31 | 13.0 | 8.0 | 2.5 | 5.0 | 2.0 |
| 32 | LB-32 | 9.0 | 5.0 | 7.0 | 8.5 | 2.0 |

**Supplementary Table S4a:** Real-time absolute quantification of leaf blight/spot blotch isolates population in the rhizosphere of susceptible wheat variety Sonalika at 15-day intervals for a period of 3 months (*Unit: 1 × 10^8^ DNA copy number)

| S.No | Isolate | I | II | III | IV | V |
| --- | --- | --- | --- | --- | --- | --- |
| 1 | LB-1 | 70.55 | 267.49 | 473.00 | 1345.58 | 1735.14 |
| 2 | LB-2 | 99.67 | 124.83 | 354.05 | 1235.01 | 70.02 |
| 3 | LB-3 | 55.32 | 283.89 | 601.38 | 1409.79 | 144.80 |
| 4 | LB-4 | 56.41 | 127.19 | 240.91 | 266.34 | 456.46 |
| 5 | LB-5 | 55.32 | 521.11 | 1388.92 | 2814.06 | 659.18 |
| 6 | LB-6 | 136.51 | 245.15 | 711.77 | 1020.12 | 2782.40 |
| 7 | LB-7 | 127.66 | 527.16 | 1428.30 | 2536.21 | 1852.99 |
| 8 | LB-8 | 57.94 | 199.22 | 229.12 | 524.35 | 4.74 |
| 9 | LB-9 | 257.82 | 246.49 | 816.83 | 1691.17 | 502.03 |
| 10 | LB-10 | 71.04 | 111.34 | 407.63 | 1283.31 | 41.29 |
| 11 | LB-11 | 10.35 | 117.42 | 353.50 | 225.88 | 42.43 |
| 12 | LB-12 | 232.96 | 305.69 | 565.88 | 3207.69 | 1.22 |
| 13 | LB-13 | 139.23 | 225.99 | 619.81 | 2784.96 | 530.44 |
| 14 | LB-14 | 23.07 | 124.87 | 161.89 | 127.46 | 121.82 |
| 15 | LB-15 | 49.77 | 294.09 | 1109.03 | 1209.02 | 2914.52 |
| 16 | LB-16 | 105.78 | 134.18 | 522.37 | 610.87 | 14.67 |
| 17 | LB-17 | 133.58 | 500.44 | 597.22 | 1210.19 | 36.10 |
| 18 | LB-18 | 101.70 | 115.49 | 145.17 | 178.83 | 19.18 |
| 19 | LB-19 | 137.14 | 437.70 | 1205.64 | 2701.50 | 45.69 |
| 20 | LB-20 | 25.74 | 127.63 | 679.03 | 1840.95 | 94.88 |
| 21 | LB-21 | 80.27 | 234.94 | 657.33 | 2124.43 | 395.46 |
| 22 | LB-22 | 22.03 | 60.64 | 139.86 | 693.22 | 12.48 |
| 23 | LB-23 | 23.46 | 54.13 | 126.00 | 227.02 | 4.48 |
| 24 | LB-24 | 116.00 | 247.24 | 1090.58 | 1740.07 | 5.08 |
| 25 | LB-25 | 39.39 | 57.91 | 31.89 | 21.86 | 19.94 |
| 26 | LB-26 | 23.87 | 235.01 | 578.36 | 714.06 | 71.43 |
| 27 | LB-27 | 48.98 | 397.72 | 691.20 | 1474.69 | 493.62 |
| 28 | LB-28 | 52.82 | 227.07 | 449.89 | 571.91 | 23.53 |
| 29 | LB-29 | 126.84 | 712.37 | 962.57 | 2997.33 | 228.25 |
| 30 | LB-30 | 113.04 | 189.23 | 266.99 | 1114.17 | 31.76 |
| 31 | LB-31 | 42.65 | 127.21 | 333.74 | 620.59 | 13.47 |
| 32 | LB-32 | 27.05 | 479.48 | 572.01 | 838.30 | 8.96 |

**Supplementary Table S4b:** Real-time absolute quantification of leaf blight/spot blotch isolates population in the rhizosphere of moderately resistant wheat variety HD2733 at 15-day intervals for a period of 3 months (*Unit: 1 × 10^8^ DNA copy number)

| S.No | Isolate | I | II | III | IV | V |
| --- | --- | --- | --- | --- | --- | --- |
| 1 | LB-1 | 53.13 | 84.87 | 172.79 | 418.63 | 601.32 |
| 2 | LB-2 | 66.70 | 104.61 | 245.31 | 473.39 | 47.42 |
| 3 | LB-3 | 43.60 | 114.70 | 267.38 | 556.18 | 66.21 |
| 4 | LB-4 | 39.30 | 114.81 | 166.21 | 287.59 | 348.14 |
| 5 | LB-5 | 28.81 | 99.67 | 358.08 | 476.04 | 267.52 |
| 6 | LB-6 | 31.98 | 99.87 | 117.84 | 211.42 | 334.32 |
| 7 | LB-7 | 55.22 | 263.22 | 442.13 | 247.26 | 208.98 |
| 8 | LB-8 | 23.93 | 117.82 | 258.90 | 281.43 | 12.54 |
| 9 | LB-9 | 55.10 | 232.46 | 326.90 | 422.43 | 8.13 |
| 10 | LB-10 | 98.97 | 267.46 | 505.47 | 803.68 | 512.21 |
| 11 | LB-11 | 26.20 | 127.13 | 229.68 | 569.23 | 4.59 |
| 12 | LB-12 | 15.06 | 28.76 | 283.91 | 1148.64 | 18.62 |
| 13 | LB-13 | 22.03 | 155.78 | 253.26 | 450.07 | 4.40 |
| 14 | LB-14 | 18.74 | 62.83 | 117.85 | 224.84 | 2.32 |
| 15 | LB-15 | 42.86 | 129.02 | 194.66 | 501.76 | 555.95 |
| 16 | LB-16 | 122.18 | 181.35 | 317.47 | 590.57 | 39.00 |
| 17 | LB-17 | 28.05 | 208.80 | 527.16 | 619.82 | 46.12 |
| 18 | LB-18 | 85.74 | 150.80 | 200.62 | 212.29 | 9.41 |
| 19 | LB-19 | 21.75 | 67.85 | 269.48 | 414.21 | 26.08 |
| 20 | LB-20 | 25.49 | 67.27 | 317.45 | 822.46 | 36.96 |
| 21 | LB-21 | 55.73 | 269.41 | 601.46 | 649.68 | 176.69 |
| 22 | LB-22 | 42.88 | 50.21 | 211.58 | 473.77 | 33.37 |
| 23 | LB-23 | 14.94 | 49.10 | 69.51 | 126.08 | 1.92 |
| 24 | LB-24 | 57.12 | 185.28 | 536.37 | 939.48 | 543.27 |
| 25 | LB-25 | 23.97 | 100.01 | 73.94 | 57.79 | 3.42 |
| 26 | LB-26 | 93.41 | 232.55 | 413.87 | 613.18 | 143.00 |
| 27 | LB-27 | 64.56 | 234.35 | 397.82 | 633.72 | 31.32 |
| 28 | LB-28 | 54.63 | 285.86 | 380.06 | 475.61 | 16.49 |
| 29 | LB-29 | 135.25 | 250.16 | 789.63 | 1473.17 | 49.01 |
| 30 | LB-30 | 60.59 | 164.72 | 211.93 | 294.63 | 123.04 |
| 31 | LB-31 | 26.37 | 53.30 | 167.99 | 316.47 | 20.99 |
| 32 | LB-32 | 16.93 | 48.96 | 226.21 | 332.44 | 1.52 |
